# Patient-specific driver gene prediction and risk assessment through integrated network analysis of cancer omics profiles

**DOI:** 10.1093/nar/gku1393

**Published:** 2015-01-08

**Authors:** Denis Bertrand, Kern Rei Chng, Faranak Ghazi Sherbaf, Anja Kiesel, Burton K. H. Chia, Yee Yen Sia, Sharon K. Huang, Dave S.B. Hoon, Edison T. Liu, Axel Hillmer, Niranjan Nagarajan

**Affiliations:** 1 Computational and Systems Biology, Genome Institute of Singapore, Singapore 138672, Singapore; 2 Cancer Therapeutics and Stratified Oncology, Genome Institute of Singapore, Singapore 138672, Singapore; 3 Department of Molecular Oncology, John Wayne Cancer Institute, Santa Monica, CA 90404, USA; 4 The Jackson Laboratory for Genomic Medicine, Farmington, CT 06030, USA

## Abstract

Extensive and multi-dimensional data sets generated from recent cancer omics profiling projects have presented new challenges and opportunities for unraveling the complexity of cancer genome landscapes. In particular, distinguishing the unique complement of genes that drive tumorigenesis in each patient from a sea of passenger mutations is necessary for translating the full benefit of cancer genome sequencing into the clinic. We address this need by presenting a data integration framework (OncoIMPACT) to nominate patient-specific driver genes based on their phenotypic impact. Extensive *in silico* and *in vitro* validation helped establish OncoIMPACT's robustness, improved precision over competing approaches and verifiable patient and cell line specific predictions (2/2 and 6/7 true positives and negatives, respectively). In particular, we computationally predicted and experimentally validated the gene *TRIM24* as a putative novel amplified driver in a melanoma patient. Applying OncoIMPACT to more than 1000 tumor samples, we generated patient-specific driver gene lists in five different cancer types to identify modes of synergistic action. We also provide the first demonstration that computationally derived driver mutation signatures can be overall superior to single gene and gene expression based signatures in enabling patient stratification and prognostication. Source code and executables for OncoIMPACT are freely available from http://sourceforge.net/projects/oncoimpact.

## INTRODUCTION

In recent years, advances in genomic technologies have enabled the systematic generation of clinical cancer omics data at an unprecedented scale and rate, interrogating tumor biology at multiple levels—genomic, transcriptomic as well as epigenomic (1,2). Integrative mining of these clinically characterized, information-rich data sets is expected to provide deep insights into tumor biology and guide new efforts to develop cancer diagnostics and therapeutics (3,4). Recent studies have, however, highlighted the complexity of cancer genome landscapes in terms of somatic mutations, transcriptomic changes and epigenetic alterations, potentially confounding modeling, mining and integrative analysis of cancer omics data (5,6). While the complexity of cellular processes that link the different levels of changes in cancer cells may suggest the use of sophisticated systems biology (mechanistic or probabilistic) models for data integration, their utility can be hampered by the need to learn a large number of parameters from a limited number of patient samples (7). On the other hand, it is unclear if simpler models can adequately capture key features of the data and be used to obtain biologically relevant insights. Correspondingly, despite its importance, relatively few methods have been proposed that can model and integrate cancer omics data (8–11) and limitations in mining and interpretation continue to be a major barrier for their exploitation in clinical applications (3,12).

One of the fundamental challenges in the analysis and interpretation of cancer genomic data is to identify and distinguish functional (driver) mutations from the numerous non-functional (passenger) mutations that are found to populate cancer genomes (13,14). This problem has relevance not only for an understanding of tumor biology (in terms of characterizing oncogenes and tumor suppressors) but also from a clinical perspective where patient-specific driver genes hold significant value for defining therapeutic targets. While recent studies that have cataloged the frequency of mutations in genes based on a large number of patient samples have been quite successful in identifying the major oncogenes and tumor suppressors in a cancer subtype (15,16), these approaches are not well-suited for identifying rare drivers or patient-specific driver genes (14,17), even with the use of more sophisticated statistical approaches (18,19). An orthogonal approach that has been used with some success relies on the direct evaluation of evolutionary conservation and physiochemical properties to infer functional mutations (20,21), but these methods are restricted to point mutations and were found to lack in accuracy due to a dependence on high-quality training data (22). Integration of mutation data with gene interaction networks has also been proposed as an approach to identify rare drivers, relying on the assumption that they will cluster on the network, but limited to the analysis of point mutations (23–25).

A natural and powerful approach to assess the functional impact of mutations is to measure changes in gene expression patterns that can be attributed to them. When done without prior information about which genes interact, this association analysis requires a large number of samples and can potentially lead to many false positives (9,17). Alternatively, reconstructed interaction networks based on gene co-expression (26) or known molecular networks (8,10) have been exploited to better define informative associations. These methods come closer to integrative modeling of cancer omics data and have the potential advantage of providing biologically plausible hypotheses for candidate driver genes. In addition, these methods can be applied to a range of mutation classes, unlike several popular mutation-type restricted methods (e.g. CHASM (20), OncodriveFM (18) and PARADIGM-SHIFT (11)), thus allowing for a joint assessment of driver events and genes. They are, however, currently still limited to making aggregate predictions for a data set and are not designed to support the sample-specific analysis that would be key for defining personalized cancer management and therapy. An additional limitation in the field is that existing methods have not been shown to robustly analyze data from cancer cell lines, which are frequently used as *in vitro* models for pharmacological investigations (3) and can form the basis of a framework for personalized cancer therapy.

Tumor stratification and prognostication is another important end-goal for cancer genomic profiling and analysis (27) that is often considered independent of driver gene prediction, despite being potentially related objectives. A commonly used approach for tumor stratification is based on the clustering of gene expression profiles, even though its prognostic value has appeared limited at times and depends greatly on the adopted gene signature (28). Improved driver gene prediction should, in principle, be informative for tumor stratification as the identified mutated genes are likely causative events for carcinogenesis and metastasis. However, to our knowledge, this application has yet to be demonstrated by driver gene prediction algorithms, despite a report on whole-exome mutation profiles being useful for tumor stratification (29).

Advances in the capability to identify oncogenic drivers and to stratify tumors can potentially revolutionize personalized cancer therapy (3,27). To address existing methodological limitations, we developed a first-in-class algorithmic framework (OncoIMPACT) that nominates *patient-specifi*c *driver genes* by integratively modeling genomic mutations (point, structural and copy-number) and the resulting perturbations in transcriptional programs via defined molecular networks. Our benchmark analysis on large publicly available data sets from The Cancer Genome Atlas (TCGA) for several cancer subtypes revealed notable improvements over existing approaches in terms of precision and robustness for identifying driver genes. Furthermore, OncoIMPACT's robustness on cell line data sets was confirmed using data from the Cancer Cell Line Encyclopedia (CCLE) (30) and we additionally provide direct experimental evidence using a patient-derived cancer cell line to showcase its potential in personalized medicine. Finally, we present the first demonstration for the use of a set of computationally identified driver genes as a mutational-status-based signature for tumor stratification and prognostication. Taken together, our results highlight the potential of computational methods in integrative modeling of cancer omics data for uncovering new insights into tumor biology, and their application in a clinical setting for stratification and personalized therapy.

## MATERIALS AND METHODS

### Design of a robust framework for patient-specific data integration

A natural framework to assess the impact of candidate driver mutations (genomic and epigenomic) is to use gene interaction networks to associate mutations with changes in cell state (e.g. transcriptome (8), proteome, epigenome or metabolome) and this is the approach adopted in the design of OncoIMPACT. For the sake of simplicity and due to its wide availability, we consider only transcriptomic changes in this study, though similar ideas as proposed here apply to other omics information as well. A key consideration in the design of OncoIMPACT is the ability to characterize the *impact* of mutations (non-synonymous Single Nucleotide Variantions (SNV), indels and Copy Number Variations (CNV)) at a patient-specific level and for that purpose we propose an approach that associates mutations with modules of patient-specific deregulated genes on the network. Specifically, given a mutation in a patient we consider a deregulated gene in the patient as being *explained* if there is a small path (length less than a parameter *L*) of deregulated genes in the patient that connect it to the mutated gene in the interaction network. To account for promiscuous associations, we disallow paths that go through hub genes (with degree greater than a parameter *D*) in the network and identify *deregulated* genes as those that are significantly differentially expressed in cancer versus normal cells (false discovery rate corrected *P*-value < 0.05) and with a strong fold change (greater than a parameter *F*). The parameters in this framework (*L, D* and *F*) are directly determined using a statistical approach based on the interaction network and data sets used, as discussed in the next section. In order to cluster mutations and deregulated genes into relevant modules, we then define the notion of a *phenotype* gene as frequently explained (default ≥5% of patients) deregulated genes for a cancer subtype, where the phenotype genes serve to represent and nucleate modules as described in a later section. Finally, OncoIMPACT distinguishes passenger mutations from potential driver mutations by identifying those that explain phenotype genes and thus have a significant impact on the associated modules.

### A systematic approach to determine model parameters

While the parameters *L* and *D* are largely determined by the properties of the network, the fold-change parameter *F* could potentially interact with them to increase the number of spurious associations to a mutation. Under the assumption that with a suitable set of parameters, real data sets should have many more associations than random data sets, we use the following permutation-based approach to set parameters: (i) We generate random data sets by permuting gene labels for mutation and transcriptome data sets independently. Note that this procedure maintains the frequency distributions of mutated and deregulated genes across patients and within a patient, while destroying the association between mutated and deregulated genes. (ii) For each random data set (which contains the same number of patients as the real data set), we identify explained genes on the network and compute the distribution of the frequency with which a gene is explained. (iii) Aggregating this information across data sets, we compare it to the distribution for the real data set using the Jensen–Shannon divergence metric. (iv) A grid search over suitable ranges of parameter values is then used to set the parameters based on the choice that maximizes the Jensen–Shannon divergence from random data sets (default settings: $L \in \{ 2,4, \ldots ,20\} ,\;D \in \{ 10,15, \ldots ,100\} \;{\rm and}\;F \in \{ 1,1.5, \ldots ,3\}$). To avoid extreme parameter choices, we ignore choices for which the median number of deregulated genes (across samples) is more than half the genes in the network or less than 300 genes. Our experiments with subsets of patients confirmed the robustness of the parameter inference procedure and the feasibility to do it with small data sets to reduce overall running time (Supplementary Figure S1).

### Assessing the significance of phenotype genes

In order to identify statistically significant phenotype genes, we adopted a permutation-based testing framework to test each candidate. Specifically, we permuted gene labels for the mutations for each sample independently. The random data sets were then used to obtain an empirical null distribution (default = 500 data sets) for the frequency with which a gene is explained and compute *P*-values for observed frequencies (= probability of observing frequencies that exceed the observed frequency by chance). Corrections for multiple hypothesis testing were done using the method of Benjamini and Hochberg and a significance threshold of 0.1 was used in addition to the frequency threshold (default = 5%) to identify significant and meaningful phenotype genes for nucleating modules.

### Distinguishing driver mutations from back-seat driver mutations

While the approach to individually assess the impact of mutations and to use their association with phenotype genes for distinguishing potential drivers from passengers works reasonably well, in situations where a strong driver deregulates many genes in the network, extraneous mutated genes in the neighborhood can get associated with a module. In order to distinguish such *back-seat driver* mutations, we applied a parsimony principle to identify a minimal set of drivers associated with phenotype genes. Encoding the patient-specific association of mutations with phenotype genes as set (also implicitly defining a bipartite graph), this problem can be formulated as the classical *Minimum Set Cover* problem, a well-known NP-complete problem with a greedy *O*(log *n*) approximation algorithm. In OncoIMPACT, we implemented a version of this algorithm that iteratively selects the gene covering the most number of uncovered phenotype genes, breaking ties by choosing the gene predicted as a driver in the most number of patients. In the patient-specific mode, a mutated gene is considered as a driver in a patient only if it aided in covering a patient-specific phenotype gene (*stringent mode*), while in a more relaxed setting (*sensitive mode*; default) OncoIMPACT marks a potential driver gene as a back-seat driver only if it is so in all patients. Note that the stringent mode is particularly well suited for analyzing data sets where there is a high rate of false-positive mutations.

### Construction of patient-specific gene modules for assessing mutational impact

The construction of patient-specific gene modules in OncoIMPACT allows us to obtain a more comprehensive measure of the impact/importance of a putative driver gene. To coalesce mutated genes and phenotype genes into modules we employ the following steps: (i) For each patient a driver gene defines a module composed of the set of explained genes associated with it. (ii) Modules of the same patient that share a phenotype gene are merged together. (iii) Deregulated genes that do not belong to paths between driver and phenotype genes are trimmed from modules. (iv) The patient-specific *impact* of a driver gene is computed as the sum of fold change of genes that belong to its module and the overall *impact* is defined as the average patient-specific impact. Finally, OncoIMPACT orders predicted driver genes based on their impact value.

### Use of pre-computed information from large public data sets

OncoIMPACT is configured to run in two modes: (i) a *database* mode that allows it to determine parameter settings (*L, D* and *F*) and significant phenotype genes from the data sets provided and (ii) a *discovery* mode where information in the provided database is used to predict driver genes for each sample in an additional data set (which can be the same as the one used to create the database). In the *discovery* mode, identification of *back-seat drivers* is done by combining the *database* data sets with *discovery* data sets. Note that OncoIMPACT can be run by default in a combined *database-plus-discovery* setting on an input data set, while the *discovery* mode is useful to avoid computations when a pre-computed database is available. As part of the OncoIMPACT package, we provide databases constructed from all the TCGA data sets analyzed in this study, to enable easy integration with custom, in-house data sets for these cancer subtypes. New releases of OncoIMPACT will include additional subtype databases as well.

### Patient stratification and survival analysis

Clusterings of driver gene profiles (binary 1–0 vectors) were computed using non-negative matrix factorization (NMF) based on consensus clustering using the R package ‘nmf’ (31). In order to produce robust clustering the consensus clustering was obtain using 200 random runs of the NMF optimization algorithm. Kaplan–Meier curves were drawn for the clusters and log rank *P*-values computed using the R package ‘survival’ (29).

### Data sets and networks

All TCGA data sets were downloaded from the TCGA data portal (https://tcga-data.nci.nih.gov/tcga/). OncoIMPACT analysis was restricted to samples for which information on point mutations, copy-number alterations and gene expression was available. Cell line data sets (47 ovarian and 41 glioma lines) were downloaded from the CCLE data portal (http://www.broadinstitute.org/ccle/home) and shRNA data from the Achilles data portal (http://www.broadinstitute.org/achilles; 24 ovarian cell lines with genomic and shRNA data). A detailed description of data parsing and pre-processing steps can be found in the Supplementary Text.

By default, OncoIMPACT uses the gene interaction network constructed by Wu *et al*. (32) (covering nearly 50% of the human proteome) for its analysis. This interaction network integrates information from known pathways (e.g. KEGG, NCI-Nature) as well as interactions derived from computational predictions (e.g. gene co-expression, protein domain interactions and shared gene ontology (GO) biological process). However, OncoIMPACT can use other networks as input as well and our experiments with a manually curated network (33) suggest that while a less complete network can reduce its predictive power, its predictions are still typically better than a frequency-based approach (Supplementary Figure S2).

### Genomic analysis of melanoma sample and functional validation using patient-derived cell line

Distant metastasis melanoma samples and the corresponding patient-derived cancer cell line were provided and established by the John Wayne Cancer Institute as previously described (34). Details of genome and transcriptome sequencing and analysis of the melanoma samples can be found in the Supplementary Text. Driver genes in the cell line derived from distant metastasis were validated using siRNA-mediated knockdown. Briefly, the patient-derived cell line was cultured in complete RPMI culture medium containing 10% fetal bovine serum and was kept at 37°C with 5% CO_2_. For the knockdown experiment, cells were incubated with 25 nM siRNA and lipofectamine RNAimax (Life Technologies) at 37°C for 72 h. Active cell proliferation was detected using Click-iT EdU Alexa Fluor 488 HTS Assay (Life Technologies). Fixation and staining of cells was performed according to manufacturer's instructions. The siRNAs used are tabulated in Supplementary Table S1. Dharmacon ON-TARGETplus Non-Targeting Control Pool was used as a negative control. The TaqMan primers for quantitative polymerase chain reaction are designed by and ordered comerically from Life Technologies.

## RESULTS

### An overview of OncoIMPACT's algorithmic framework

**Figure 1.**
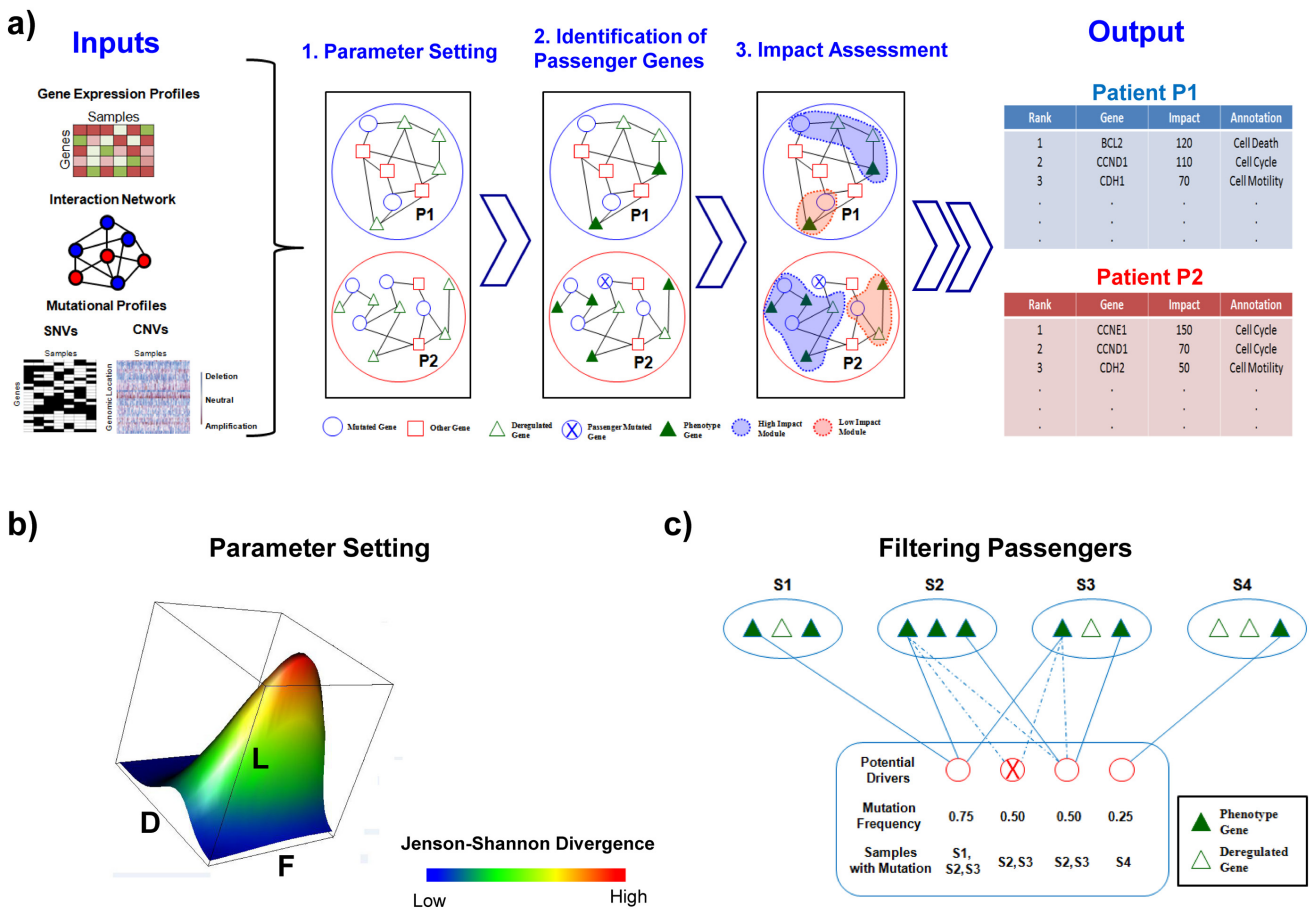
A schematic representation of OncoIMPACT's algorithmic framework. (a) Overview of OncoIMPACT's workflow involving three main stages of data-processing. (b) Depiction of OncoIMPACT's search through a multi-dimensional space to set network and expression parameters (F, fold change of genes; L, length of path; D, degree of nodes). (c) Parsimony-based matching of potential driver and phenotype genes in a bipartite graph to eliminate *back-seat* drivers. Solid and dashed lines indicate the association of potential driver genes to phenotype genes that were accepted and rejected, respectively.

OncoIMPACT is designed to integrate information regarding mutations (genomic and epigenomic), changes in cell state (e.g. transcriptome, proteome, epigenome or metabolome) and gene interaction networks to nominate and rank driver cancer mutations in a patient-specific manner (i.e. driver predictions are made for each patient; Figure 1a and Materials and Methods). Briefly, it does so by evaluating the *impact* of a mutation by associating them to modules of patient-specific deregulated genes through the gene interaction network (step 3 in Figure 1a). A key step in this process is the identification of sentinel *phenotype* genes frequently deregulated in a cancer subtype (but not typically mutated) and serve to distinguish relevant driver mutations from passengers (step 2 in Figure 1a). The association of mutations to phenotype genes is controlled by three parameters (maximum path length *L*, maximum gene connectivity *D* and a perturbation threshold *F*) that are determined in a data-driven fashion using a statistical maximization approach (step 1 in Figure 1a, b and Materials and Methods). To further differentiate true drivers from *back-seat drivers*, OncoIMPACT employs the parsimony principle to identify a minimal set of driver mutations for each patient (Figure 1c). Finally, the nominated patient-specific drivers are ranked based on their impact on associated modules. A detailed description for each of the steps in OncoIMPACT can be found in the Materials and Methods section.

### OncoIMPACT nominates cancer drivers accurately and consistently

**Figure 2.**
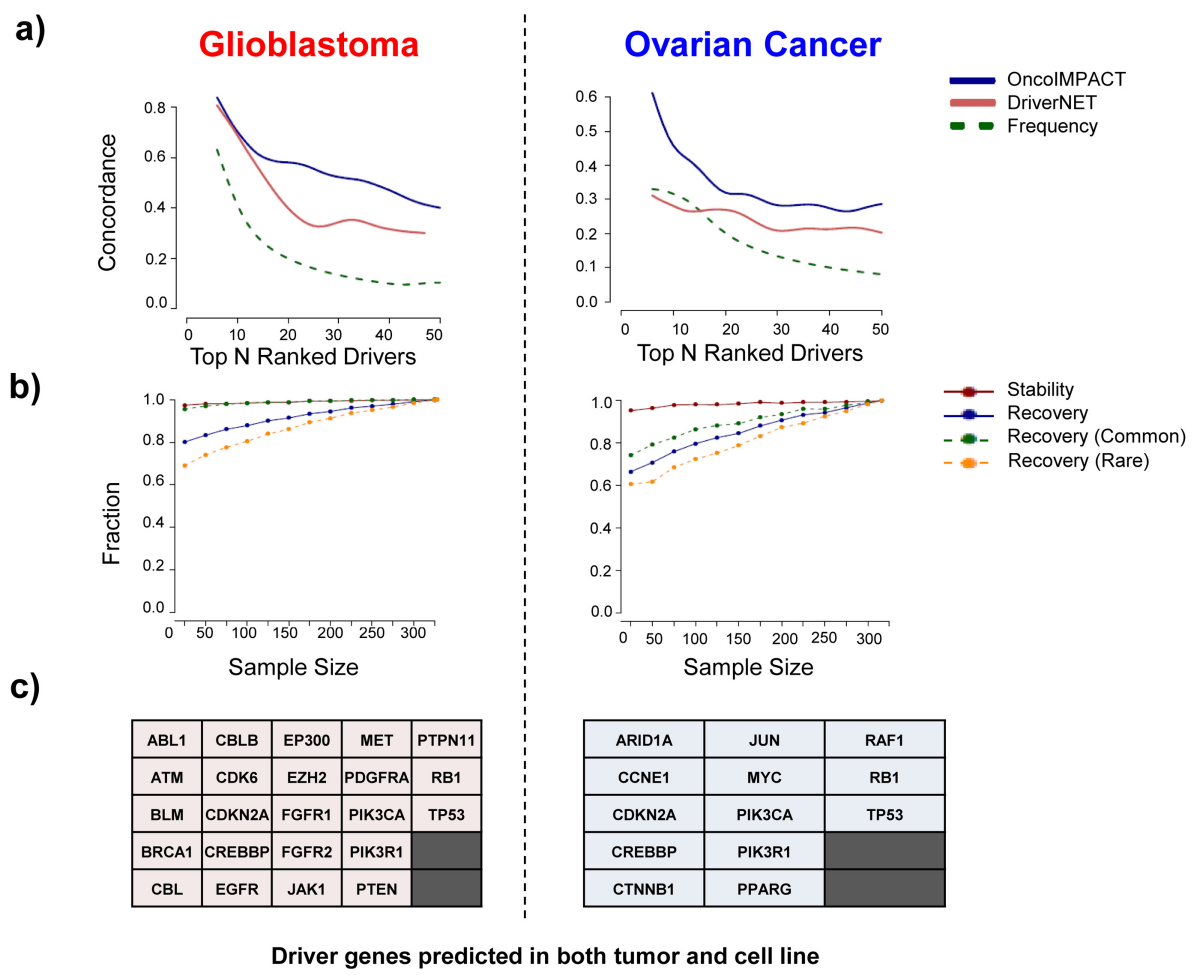
OncoIMPACT makes precise and robust driver gene predictions. (a) Concordance/precision measured by the fraction of top ranked driver genes from OncoIMPACT, DriverNet and a frequency-based approach that are included in the CGC and a pan-cancer driver gene list. (b) Stability (precision when evaluated on predictions from the full data set) and recovery (sensitivity when evaluated on the full data set) characteristics of OncoIMPACT as a function of the size of the data set analyzed (average of 20 simulations). (c) Common genes predicted as drivers by OncoIMPACT in both clinical tumor samples and cell lines (cancer census genes).

As existing methods for identifying driver genes are based on aggregate analysis over a large number of patients, we begin by comparing OncoIMPACT's performance for this task against an aggregate network approach (DriverNet (8)) as well as a commonly used mutation frequency-based approach for ordering candidate drivers (35–39) (Frequency). Our experiments using large TCGA data sets (328 samples for Glioblastoma multiforme or GBM (1) and 316 for Ovarian Cancer (40)) indicate that OncoIMPACT can successfully integrate information regarding copy-number as well as point mutations and indels to highlight key driver genes across categories (Supplementary Tables S2 and S3). In contrast, a naive frequency-based approach seems to enrich for less known cancer driver genes (Supplementary Tables S2 and S3 and Supplementary File S1), e.g. the top gene on the Glioblastoma list is *JARID1D* instead of *EGFR* and both lists omit *PIK3CA* from the top 10. While results for DriverNET were more comparable to those from OncoIMPACT, DriverNET failed to identify several known oncogenes, such as *NF1* and *RB1*, in ovarian cancer (40) and *MDM4* in Glioblastoma (41) among others (Supplementary Tables S4 and Supplementary File S1). To perform a more systematic comparison across methods, we used genes in the cancer gene census (CGC) (42) and a previously compiled pan-cancer driver list (43) as a proxy for potential drivers to assess the concordance/precision of the top driver genes reported for five different cancer types (GBM, Melanoma, Ovarian, Prostate and Bladder) (Figure 2a, Supplementary Figure S3). These results indicate a strong enrichment for potential true positive driver genes in OncoIMPACT's predictions (Supplementary Figure S3b). For example, among the top 20 predictions in Glioblastoma, OncoIMPACT's concordance is above 60% while the frequency-based approach and DriverNet are below 40%, suggesting that it is generally more accurate and less likely to be influenced by frequently mutated passengers. This trend was seen in all cancer types, except for the Melanoma data set where the lack of sufficient normal controls likely affected OncoIMPACT's results in relation to DriverNet (Supplementary Figure S3).

We further tested the robustness of OncoIMPACT using a subsampling-based approach to compare predictions to those on the full data set of patients. Our results suggest that OncoIMPACT's predictions are extremely stable even with very small sample sizes (∼20 patients), with more than 90% of reported drivers being found on the full data set (Figure 2b). In addition, OncoIMPACT can recover a sizable proportion of drivers using a relatively small subset of the data set (>70% with 50 patients; Figure 2b). Although both common drivers (>5% mutational frequency) and rare drivers (<5% mutational frequency) have high recovery, a higher fraction of common drivers is generally recovered, possibly due to the bias in the passenger filtering step in OncoIMPACT (Figure 2b; Materials and Methods). However, the recovery rates for rare and common drivers converge as the number of samples increases. The stability attribute of OncoIMPACT is likely to be a useful feature in the analysis of rare forms of cancers (e.g. cardiac tumors (44)), where the availability of samples is limited. In particular, the naive frequency-based approach would be unsuitable for such data sets due to limited statistics.

To further demonstrate that OncoIMPACT is able to discern true signals from noisy data, and to establish its utility for analyzing cell line data sets, we repeated our analysis using data from CCLE (30). As the cell lines here do not have normal controls, identification of somatic variants is error-prone, but despite this we found that OncoIMPACT results enrich for true drivers and are significantly better than a competing approach (Supplementary Figure S4). A significant fraction of drivers in the cell line were also further confirmed using shRNA experiments, as detailed in the next section. Cancer cell lines are commonly used as *in vitro* models for clinical tumors with the caveat that they can deviate genetically from their tumor counterparts after years of adaptation to artificial culturing conditions (45). Furthermore, there is a diversity bias inherent in cell line collections, where mutation frequencies in cell lines may not be reflective of clinical tumor collections. Despite this, our comparisons of driver genes predicted from cell lines and patient samples suggested that the overlap between them is significant (*P*-value = 2.3 × 10^−9^ and 3.3 × 10^−9^ for Glioblastoma and Ovarian Cancer, respectively; hypergeometric test, Supplementary Figure S5) and involves key known cancer driver genes (Figure 2c). However, important differences still exist in terms of cell line and patient-specific drivers (Supplementary File S2), possibly due to differences in the biological contexts in which they exist.

### OncoIMPACT makes robust and verifiable patient-specific driver gene predictions

**Figure 3.**
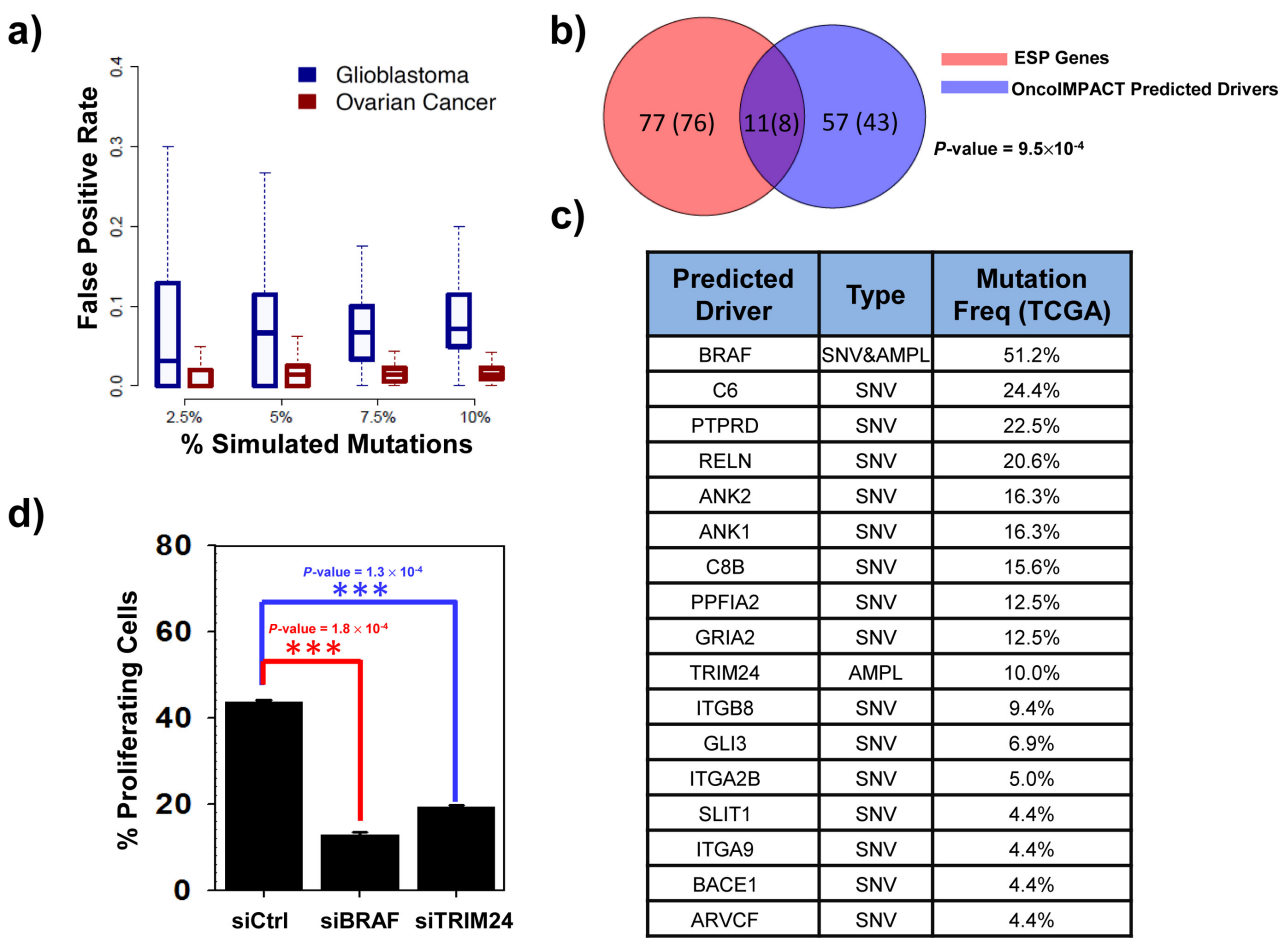
Validation of sample-specific driver gene predictions. (a) Box plots depicting the distribution across samples of FPR for driver gene predictions in OncoIMPACT (average of 20 simulations). Decoy mutations were introduced in random genes as proxy for non-drivers in this assessment. (b) Overlap between predicted unique cell line-specific drivers and shRNA validated genes (using at least 2 shRNAs) ESP in 24 ovarian cancer cell lines. Number in parenthesis represent the number of unique genes. The *P*-value is computed using hypergeometric test. (c) Frequency in TCGA samples and mutation type for driver gene predictions from a melanoma sample. (d) Cell proliferation assay in a patient-derived melanoma cell line treated with control siRNA or siRNA targeting BRAF and TRIM24. Error bars represent SEM of three independent repeats. Statistical significance was assessed by using student's *t*-test.

While the identification of patient-specific driver genes is challenging, validating a methodology that identifies them is even more so, given the lack of gold standards (e.g. by their very definition patient-specific drivers are less likely to be in CGC). We attempted to verify OncoIMPACT's ability to call patient-specific drivers using three different approaches. First, we experimented with *in silico* data sets derived from real TCGA data sets by introducing random mutations to test our ability to discriminate them. These experiments highlight that OncoIMPACT shows a high-degree of tolerance to the introduction of decoy mutations, and can robustly accommodate up to 10% of erroneous mutation data (e.g. due to sequencing or variant-calling errors) (Figure 3a, Supplementary Figure S6). In doing so, it is able to control the false positive rate (FPR) to be generally less than 10% (median FPR < 5% for 2.5% decoy mutations), suggesting that a majority of patient-specific driver predictions are likely to be true positives. To further validate the consistency of patient-specific driver gene predictions, we experimented with learning phenotype genes from a random subset of samples, with prediction on unselected samples (cross-validation). Our results show good predictive stability for all drivers and good predictive recovery for both common (>5% frequency) and rare drivers (<5% frequency) (Supplementary Figure S7), further confirming OncoIMPACT's robustness for patient-specific driver gene prediction.

Second, we leveraged data from a recent genome-scale functional screen for genes essential for survival and proliferation (ESP) in 24 different ovarian cancer cell lines (46), to evaluate our cell line-specific predictions. We noted a significant overlap (*P*-value = 9.5 × 10^−4^; hypergeometric test, Figure 3b) between predicted drivers and validated ESP genes, including several that are rarely mutated in ovarian cancer (e.g. *MAPK1* (1.6%) and *JUN* (2.2%)) and with functions consistent with cancer driver genes (Supplementary Tables S5). As control, we evaluated overlap with the frequency-based approach and found it to be not significant (*P*-value = 0.96 at frequency cutoff of 5%; hypergeometric test). Furthermore, increasing the stringency for ESP genes (validated by 4 shRNAs), increased the enrichment in OncoIMPACT predictions (5 out of 7; *P*-value = 2.5 × 10^−6^; hypergeometric test) suggesting that most strong proliferation drivers are identified by OncoIMPACT. Further validation would be needed to characterize drivers that play a role in other cancer processes, such as invasion, genome stability and angiogenesis.

Finally, to showcase OncoIMPACT's ability to combine large publicly available data sets with data from custom sample collections, we analyzed data from a clinical distant metastasis sample (paired tumor-normal exome sequencing, mate-pair sequencing-based copy-number profiling and RNA-seq) in combination with a data set of 160 melanoma samples from TCGA (see Materials and Methods). Despite the presence of a large number of mutations and amplifications in the sample, OncoIMPACT nominated a concise list of driver mutations (Figure 3c). As proof of principle, a patient-derived cell line from the patient was used for experimental validation (see Materials and Methods). Using siRNAs, we attempted to knock-down the expression of the two amplified, predicted driver genes in the cell line (*BRAF* and *TRIM24*, Supplementary Figure S8) and noted a substantial reduction in the proliferation rate of cancer cells for both genes (Figure 3d, Supplementary Figure S9). *BRAF* is a well-known frequently mutated and amplified driver in melanoma (47). On the other hand, even though *TRIM24-BRAF* fusion gene was previously reported to be present in a subset of melanomas (48), *TRIM24* on its own has not been characterized as a driver and was ranked 671 in the list of frequently mutated genes in the TCGA melanoma data set. Interestingly, while *TRIM24* was initially identified as a transcriptional co-regulator, it has been recently shown to ubiquitinate *TP53* for degradation in breast cancer (49) and could play a similar role in melanoma. We further performed an experimental assessment of our false-negative rate by silencing seven selected amplified genes (*CASP2, CNOT4, CUL1, EZH2, HIPK2, SSBP1* and *ZYX*) with known functions in oncogenic processes that were not predicted as drivers by OncoIMPACT (Supplementary Figure S8). Strikingly, despite our enrichment for potential false negatives, we observed a reduction in the proliferation of cancer cells in only one (EZH2) out of seven selected genes (Supplementary Figure S10). Together, these results provide evidence that a computational approach, combined with clinical genome sequencing could serve as a means to reliably identify personalized therapeutic targets in cancer.

### Distributional properties of driver genes and associated deregulated modules

**Figure 4.**
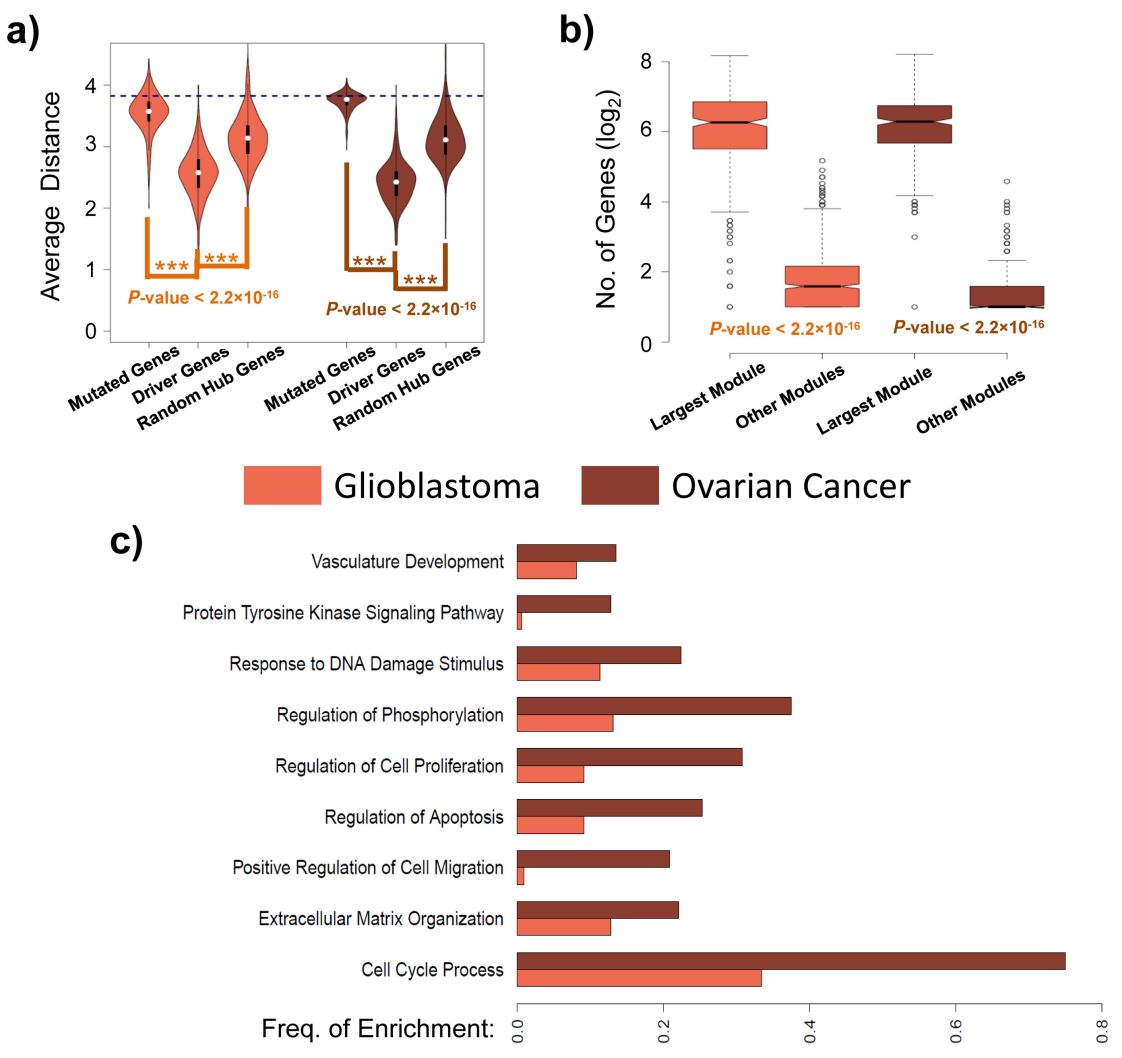
Clustering of driver genes and GO enrichment of associated modules. (a) Violin plots showing the distribution of average distance in the gene interaction network (computed at a sample-specific level) between all pairs of genes in each class (mutated genes, predicted driver genes and random hub genes (degree ≥ 20)). The blue line represents the average distance between genes on the interaction network. The *P*-values are computed using Wilcoxon rank sum test. (b) Box plots depicting the distribution of the number of genes in the largest module and all other modules. The *P*-values are computed using Wilcoxon rank sum test. (c) Bar chart showing the frequency at which GO terms are enriched in the largest module for each patient. Enrichment analysis was done using DAVID (53) (http://david.abcc.ncifcrf.gov/) and a *q*-value threshold of 0.05 was used to identify enriched terms.

The ability to generate patient-specific lists of driver genes allowed us to analyze the distributional properties of driver genes without having to resort to an aggregate analysis that may obscure its interpretation. For example, using all predicted drivers, we readily observed that driver genes tend to cluster on the gene interaction network, similar to what was observed by others (50), and distances between them were significantly lower than between all mutated genes and between random genes (Supplementary Figure S11). However, this observation has several potential explanations including, but not limited to: (i) tumors share driver mutations that affect the same functional network (29) and (ii) biases in the data (50). Analysis using patient-specific driver gene lists avoids some of these issues and our analysis using OncoIMPACT revealed a similar pattern of clustering at a sample-specific level (Figure 4a and Supplementary Figure S12), that is not explained by mutation frequency or network structure (i.e. hub genes), suggesting that an alternative explanation—that the occurrence of multiple mutations in a network module is necessary for pathway deregulation in a tumor—may be valid here. We further investigated such synergistic interactions between drivers by using an unbiased search and statistical testing to identify potential co-drivers and compared our patient-specific results with a non-patient-specific approach (Supplementary Figure S13, Supplementary File S3). Our results show that the patient-specific analysis likely identifies more meaningful co-driver gene pairs (i) identifying a smaller subset of potential gene-pairs as co-drivers (Supplementary Figure S13a), (ii) that are less likely to be enriched in false-positives due to genomic proximity (Supplementary Figure S13b) and (iii) are more enriched in genes that are likely to have similar functional roles (Supplementary Figure S13c). In addition, we identified several gene-pairs as co-drivers that were not necessarily correlated in their mutation occurrences and were not therefore detectable without a patient-specific analysis as provided by OncoIMPACT (Supplementary File S3). Interestingly, in comparison to glioblastoma, ovarian and prostate cancer, we noted only a handful of co-drivers in melanoma and bladder cancer (Supplementary Figure S13a) and we discuss this observation further in a following section (see Discussion).

An intrinsic feature of OncoIMPACT is that it ‘annotates’ candidate driver genes with an associated module of deregulated genes and phenotype genes in the network that can provide hints to the mechanism by which the putative driver acts as one (see Materials and Methods). For example, in our analysis of the TCGA Ovarian Cancer data set, amplification of the *c-MYC* oncogene was frequently associated (in 58% of mutated tumors) with an increase in the expression of the phenotype gene *BCAT1*, an amino acid transaminase that produces branched-chain L-amino acids required for cell proliferation. This suggests that *BCAT1* may be a direct transcriptional target of *c-MYC* and an effector through which *c-MYC* exerts its oncogenic influence, a relationship that has previously been demonstrated in nasopharyngeal carcinoma (51), but needs to be explored in ovarian cancers. As another notable example, we observed that the amplification of *DVL3* was associated in 81% of tumors by a corresponding down-regulation in the expression of the phenotype gene *CXXC4*. *DVL3* is a human homolog of the Drosophila dishevelled gene and to our knowledge, not yet directly implicated as a driver in ovarian cancers. *CXXC4* is known to negatively regulate the Wnt signaling pathway by binding and inhibiting Dvl (52). Thus, our analysis suggests the hypothesis that *DVL3* amplifications, coupled with decrease in *CXXC4* expression, could drive ovarian cancer progression through enhanced activation of the Wnt signaling pathway. These and other novel driver gene predictions from OncoIMPACT can be further investigated based on downstream analysis (e.g. GO enrichment) and *in vitro* testing of the phenotype genes and modules associated with them.

We further analyzed the patient-specific deregulated modules from OncoIMPACT and observed that multiple distinct deregulated modules exist in most patients (Supplementary Figure S14). Despite this, a single dominant module comprising of multiple driver genes was also frequently observed (Figure 4b and Supplementary Figure S15), suggestive of the existence of a core deregulated module driving cancer progression in each tumor sample. Furthermore, GO enrichment analysis (53) of the dominant modules showed that they are frequently enriched for genes that regulate cellular processes contributing to the hallmarks of cancer (54) (e.g. regulation of cell proliferation, apoptosis and cell migration; Figure 4c). The functional enrichment of deregulated modules in these processes further demonstrates their utility for guiding validation (e.g. distinguishing proliferation drivers from angiogenesis drivers) and mechanism-informed therapy from patient-specific predictions in OncoIMPACT.

### Tumor stratification using personalized driver mutation profiles

**Figure 5.**
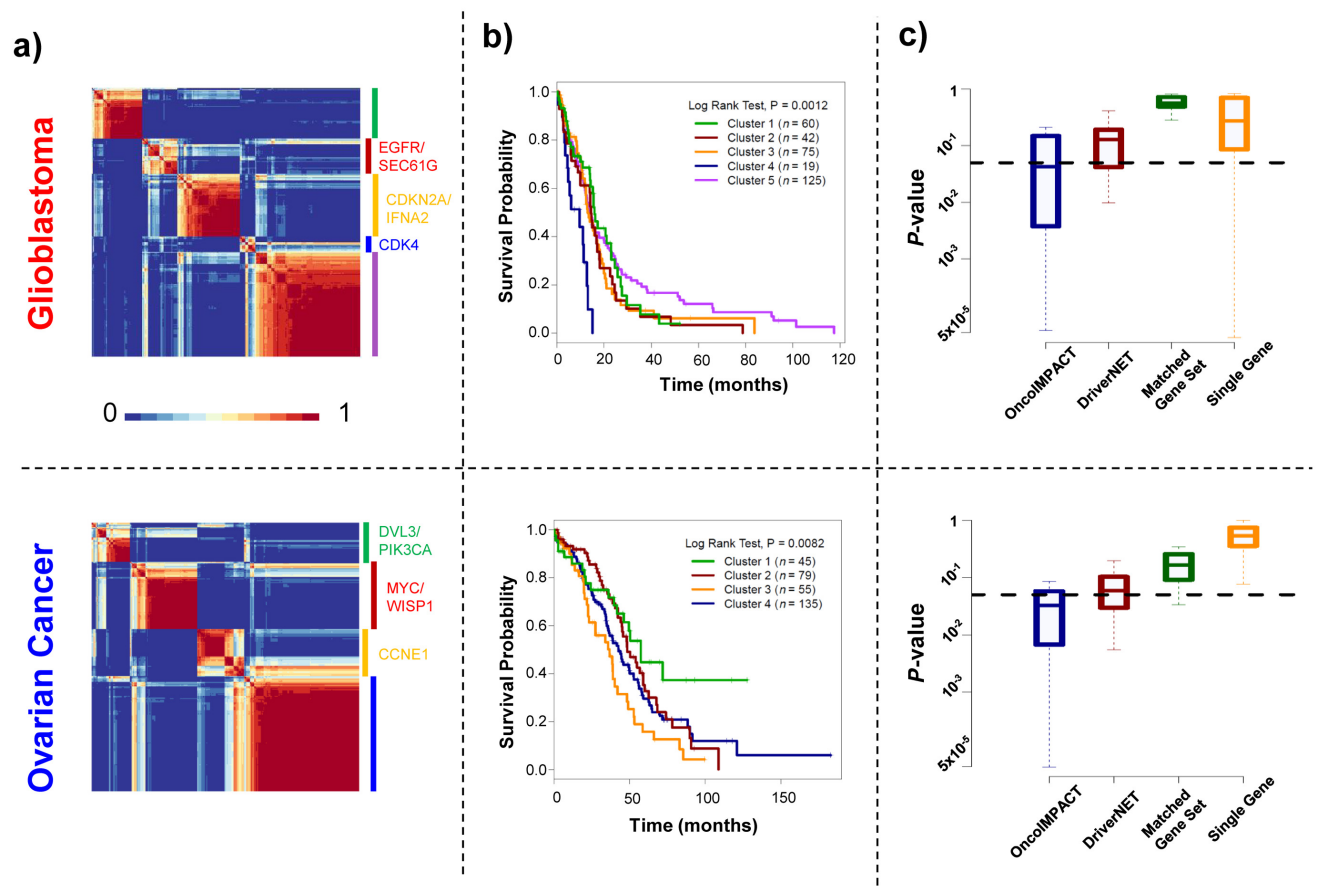
Tumor stratification using predicted driver gene profiles. (a) Heatmaps depicting consistency of clustering (fraction of bootstrap replicates in which patients clustered together) for predicted driver gene mutational profiles (binary 0–1 vectors) using NMF. (b) Survival profiles of glioblastoma and ovarian cancer patients stratified by consensus clustering in (a). (c) Box plots showing the distribution of *P*-values (log rank test) for survival profiles of random subsets of glioblastoma (sample size 275) and ovarian cancer (sample size 250) patients, clustered into the same number of groups using different gene signatures (OncoIMPACT predicted driver genes; DriverNET predicted driver genes; Randomly selected sets of genes of the same size as OncoIMPACT predicted drivers; Randomly selected single genes).

Patient-specific driver mutational profiles are potentially promising inputs for tumor stratification since by definition, they are likely causative events for carcinogenesis and metastasis. However, while the mutational status of selected single genes has been shown to be of value in various cancers (16,55–56), unsupervised stratification using whole-exome mutation profiles is significantly more challenging (29), and the use of a small, computationally derived driver gene list for this purpose has not been demonstrated before. As a first, pilot exploration of this concept, we tested the utility of OncoIMPACT's predictions for stratifying patients according to their survival outcomes. Specifically, we used unsupervised consensus clustering using NMF to cluster patient-specific driver mutational profiles. Despite the sparseness of mutational profiles and the use of only a subset of genes containing predicted driver mutations (307 and 183 genes for Glioblastoma and Ovarian Cancer, respectively), we obtained robust clustering of patients (Figure 5a). In addition, we found that most clusters are defined by a few key driver genes that are predominantly mutated in tumors belonging to that cluster and serve to distinguish them from tumors in other clusters (Figure 5a).

Evaluation of survival outcomes for patients in these clusters using Kaplan–Meier statistics suggested that the clusters carry significant prognostic value for survival (Figure 5b). For example, for Glioblastoma, cluster 4 has a mean survival time of 7.3 months compared to cluster 5 with a mean survival time of 18.4 months. Further analysis of these prognostic driver mutation signatures suggested that a smaller subset of them (using as few as the top 47 and top 6 genes for Glioblastoma and Ovarian Cancer, respectively) could contribute to the development of clinical grade signatures (Supplementary Figure S16). Overall, gene signatures selected based on driver genes also showed significantly better prognostic value compared to similar sized subsets of genes selected from all mutated genes, as well as single gene classifiers (Figure 5c). Comparison with mRNA expression profiles suggested that driver mutation-based stratification could provide better patient survival predictive value overall (Supplementary Figure S17). This could also extend to other attributes, e.g. in prostate cancer this approach successfully clustered patients into subgroups with differential prostate-specific antigen expression, the main biomarker for prostate cancer (Supplementary Figure S18). In all, these results not only highlight the promise of driver mutation profiles to stratify patients in an unsupervised fashion, but also indirectly confirm the quality of driver gene predictions from OncoIMPACT.

## DISCUSSION

In recent years, as the generation of high-dimensional molecular profiling data sets has become easier, the challenge has naturally shifted toward better mining of this information. While, in principle, these data sets represent a rich resource, their high-dimensionality entails that correlations and associations are easy to find, but validating them may not be so. In particular, cancer genomics is an area that has benefitted from the facility of data generation and the focus has now rightly shifted toward integrative analysis, with driver gene identification being a key focus (57). In this work, we show that a simple, model-based approach can reliably sort through the sea of passenger mutations that dot cancer genomes (13) to nominate patient-specific drivers, outperforming competing approaches to do so. Our benchmarking analysis suggests that this approach is robust to noise and works with small data sets, making it applicable to a wider array of sample collections, including cell lines and xenograft models. By being model based, our approach distinguishes itself from others in that it can (i) provide insights into the mechanisms by which putative drivers act and (ii) enable integration of diverse molecular profiles. For example, phospho-proteomic, microRNA and methylation profiling can provide valuable additional information about tumor biology and can naturally be integrated into OncoIMPACT's model as new perturbations, genes and mutations, respectively.

The ability to make patient-specific driver predictions open up new avenues in personalized medicine and targeted cancer therapy. Our validation results suggest that OncoIMPACT is the first method to make robust and verifiable patient-specific driver gene predictions. As a proof of principle, using genomic profiling of melanoma samples and a patient-derived cell line, we provide evidence that a computational approach, such as OncoIMPACT, combined with clinical genome sequencing can help identify personalized therapeutic targets. This is an exciting area for further work, including in the development of gold standard data sets for benchmarking, and further validation to bring this vision closer to clinical practice.

Additionally, our analysis of OncoIMPACT's patient-specific driver predictions revealed new biological insights, including the clustering of driver mutations on the network for each patient, suggesting that multiple hits may be required to significantly deregulate a pathway for oncogenesis. These observations support a model where there is substantial functional redundancy between genes, giving rise to robust cellular networks. They also suggest an additional aspect to the multiple-mutation theory of carcinogenesis (58), where the functional relationship of potential driver genes (reflected by their proximity in the molecular network) may be another determining factor for progression to cancer. Further work will be needed to explore and confirm this hypothesis, particularly through single tumor cell profiling to eliminate the caveat that the identified drivers mutations may not have occurred in the same cell.

A surprising observation from our co-driver analysis was the lower number of co-drivers found in melanoma and bladder cancer compared to glioblastoma, ovarian and prostate cancer. As our statistical analysis is influenced by the frequency of driver genes, it is not clear if this observation can be solely attributed to the biological differences in specific cancer types. In addition, as co-drivers can more easily emerge from a single CNV event, this could partially explain the imbalance in the number of co-drivers observed in various cancer types. This question deserves further investigation through the analysis of additional cancer types.

Given that mutations in driver genes are the putative underlying causes for tumorigenesis and have been shown to hold strong prognostic value individually (59), driver gene prediction-based tumor stratification has been surprisingly difficult and elusive. While individual genes are useful for prognostication, a more comprehensive panel of driver genes for a cancer type can help capture relevant interactions between drivers, without sequencing the whole genome. In this study, we directly confirmed that tumors can be robustly stratified into subgroups through standard consensus clustering of digital mutational profiles restricted to driver genes predicted by OncoIMPACT. Moreover, the subgroups obtained exhibited significantly different survival outcomes, establishing the clinical relevance for such a stratification and indirect validation for OncoIMPACT's predictions. Given that DNA-based assays can be easier to work with, refinement of the mutational signatures identified here and validation using large, independent cohorts can help complement the ongoing efforts to develop RNA-based prognostic signatures (60,61).

## SUPPLEMENTARY DATA

Supplementary Data are available at NAR Online.

SUPPLEMENTARY DATA
