# Supplementary material for: Patient-specific driver gene prediction and risk assessment through integrated network analysis of cancer omics profiles
Source: Nucleic Acids Res. 2015 Jan 8;43(7):e44. doi: 10.1093/nar/gku1393 (PMC4402507; doi:10.1093/nar/gku1393)
Supplement: SUPPLEMENTARY DATA [file supp_43_7_e44__index.html]

Patient-specific driver gene prediction and risk assessment through integrated network analysis of cancer omics profiles — Patient-specific driver gene prediction and risk assessment through integrated network analysis of cancer omics profiles — SUPPLEMENTARY DATA 

# Patient-specific driver gene prediction and risk assessment through integrated network analysis of cancer omics profiles

## SUPPLEMENTARY DATA

**Files in this Data Supplement:**

- SUPPLEMENTARY DATA
- SUPPLEMENTARY DATA
- SUPPLEMENTARY DATA
- SUPPLEMENTARY DATA
- SUPPLEMENTARY DATA
