# Supplementary material for: Patient-specific driver gene prediction and risk assessment through integrated network analysis of cancer omics profiles
Source: Nucleic Acids Res. 2015 Jan 8;43(7):e44. doi: 10.1093/nar/gku1393 (PMC4402507; doi:10.1093/nar/gku1393)
Supplement: SUPPLEMENTARY DATA [file supp_gku1393_nar-02721-met-f-2014-File007.pdf]

Supplementary Table 1

BRAF siRNA (Pool)	BRAF siRNA (Individual)
ACAGAGACCUCAAGAGUAA	CAUGAAGACCUCACAGUAA
GAAUCGGGCUGGUUUCCAA	UCAGUAAGGUACGGAGUAA
CAACAACAGGGACCAGAUA	AGACGGGACUCGAGUGAUG
GAGAUGAUCAAACUUAUAG	UUACCUGGCUCACUAACUA
**TRIM24 siRNA (Pool)**	**TRIM24 siRNA (Individual)**
GAACAUACCACGACAAGCA	GAGCAUAGAUACCAAUUUA
AGACUUAUCUAAACCAGAA	GAAGAACGCCAGUUGCUUA
CUUUAGUAAUCGAGGAUAA	GAUCAUAGAUACACUAAUC
CUUUAUAGCAAACGACUGA	UAACUGUGCCUGAUUAUUA
**EZH2 siRNA (Pool)**	**CUL1 siRNA (Pool)**
CAAAGAAUCUAGCAUCAUA	CGACAGCACUCAAAUUAAA
GAGGACGGCUUCCCAAUAA	GGUUAUAUCAGUUGUCUAA
GCUGAAGCCUCAAUGUUUA	AGACUUGGAUUUCAGCAUU
GAAUGGAAACAGCGAAGGA	CAACGAAGAGUUCAGGUUU
**HIPK2 siRNA (Pool)**	**SSBP1 siRNA (Pool)**
GAGAAUCACUCCAAUCGAA	CAACAACAAUCAUAGCUGA
AGACAGGGAUUAAGUCAAA	UGAGUGACCAGACGAAAGA
GGACAAAGACAACUAGGUU	AGACAUGAGUCCGAAACAA
GCACACACGUCAAAUCAUG	ACUAAUGAGAUGUGGCGAU
**CASP2 siRNA (Pool)**	**ZYX siRNA (Pool)**
GGAGAGUGAUGCCGGUAAA	GACAAGAACUUCCACAUGA
GAACGCACUUAUCAAGGAU	GAAUGUGGCUGUCAACGAA
GCACUGGUGUUGAGCAAUG	GACCAAGAAUGAUCCUUUC
UGACGUCCAUGUUCUAUGU	GGUGAGCAGUAUUGAUUUG
**CNOT4 siRNA (Pool)**	**Dharmacon ON-TARGETplus® Non-Targeting Control**
GUAGAUGGCAGAACACUUA	UGGUUUACAUGUCGACUAA
CCAAUUCUCUCAAUAGUAC	UGGUUUACAUGUUGUGUGA
CGUCUUUGUUGUAGGUUUA	UGGUUUACAUGUUUUCUGA
UAACCUAUAUCCGGUCAGA	UGGUUUACAUGUUUUCCUA

Table S1. Tabulation of all siRNAs used in the study.

Supplementary Table 2

a)

	Gene Name	Mut Type	Freq	Cancer Census	PubMed Results
1	TP53	SNV	94.6	CC	7916
2	MYC	AMPL	30.7	CC	17988
3	ASAP1	AMPL	30.1	-	41
4	ADCY8	AMPL	29.4	-	3
5	TG	AMPL	26.6	-	4244
6	KCNQ3	AMPL	26.6	-	8
7	SLA	AMPL	26.3	-	85
8	NDRG1	AMPL	25.9	CC	188
9	ZNF572	AMPL	25.6	-	0
10	WISP1	AMPL	25.6	-	70

b)

	Gene Name	Mut Type	Freq	Cancer Census	Impact	PubMed Results
1	TP53	SNV	94.6	CC	215.6	7916
2	MYC	AMPL	30.7	CC	69.6	17988
3	PTK2	AMPL	24.7	-	50.9	1063
4	PIK3CA	AMPL	17.1	CC	44.9	1662
5	PRKCI	AMPL	19.3	-	40.7	13
6	CCNE1	AMPL	20.3	CC	37.6	247
7	COL14A1	AMPL	22.8	-	35.7	9
8	CCN3	AMPL	20.6	-	33.1	55
9	TNFSF10	AMPL	19.6	-	28.2	3751
10	PAK2	AMPL	13.9	-	27.8	85

Table S2. Top 10 driver genes in clinical ovarian cancer samples as determined by a) a frequency-based approach and b) OncoIMPACT. DriverNET shared 3 of OncoIMPACT's top 10 predictions. PubMed results were as obtained on 01/12/2014 using the keywords "cancer" and the gene name.

Supplementary Table 3

a)

	Gene Name	Mut Type	Freq	Cancer Census	PubMed Results
**1**	JARID1D	DEL	53.0	-	3
**2**	CDKN2A	DEL	45.4	CC	8501
**3**	CDKN2B	DEL	44.5	-	738
**4**	EGFR	AMPL /SNV	41.8	CC	19960
**5**	TP53	SNV	38.4	CC	7916
**6**	PTEN	SNV	32.9	CC	6753
**7**	SEC61G	AMPL	31.7	-	7
**8**	IFNE1	DEL	26.2	-	0
**9**	IFNA1	DEL	25.6	-	11
**10**	IFNA8	DEL	25.3	-	4

b)

	Gene Name	Mut Type	Freq	Cancer Census	Impact	PubMed Results
1	EGFR	AMPL/ SNV	41.8	CC	133.6	19960
2	CDKN2A	DEL	45.4	CC	111.9	8501
3	TP53	SNV	38.4	CC	103.0	7916
4	PTEN	SNV	32.9	CC	83.5	6753
5	HLA-DRB1	AMPL	18.3	-	52.1	745
6	CDK4	AMPL	11.6	CC	38.8	3176
7	PIK3CA	SNV	9.1	CC	36.4	1662
8	SEC61G	AMPL	31.7	-	35.9	7
9	PRKY	DEL	19.5	-	34.6	5
10	PIK3R1	SNV	7.9	CC	29.7	90

Table S3. Top 10 driver genes in clinical glioblastoma samples as determined by a) a frequency-based approach and b) OncoIMPACT. DriverNET shared 7 of OncoIMPACT's top 10 predictions. PubMed results were as obtained on 01/12/2014 using the keywords “cancer” and the gene name.

Supplementary Table 4

a)

	Gene Name	Mut Type	Freq	Cancer Census
1	TP53	SNV	38.4	CC
2	EGFR	AMPL/SNV	41.8	CC
3	CDKN2A	DEL	45.4	CC
4	PTEN	SNV	32.9	CC
5	UGT2B17	DEL	9.1	-
6	PIK3CA	SNV	8.8	CC
7	CYP27B1	AMPL	11.6	-
8	PIK3R1	SNV	7.9	CC
9	IDH1	SNV	6.1	CC
10	SEC61G	AMPL	31.7	-

b)

	Gene Name	Mut Type	Freq	Cancer Census
1	TP53	SNV	94.6	CC
2	GPAA1	AMPL	25.0	-
3	PIK3CA	AMPL	17.1	CC
4	POLR2H	AMPL	15.2	-
5	PRKACA	AMPL	11.7	-
6	PTK2	AMPL	24.4	-
7	UQCRFS1	AMPL	14.2	-
8	NDUFB9	AMPL	24.7	-
9	KRAS	AMPL	10.8	CC
10	ADCY8	AMPL	29.4	-

Table S4. Top 10 DriverNET nominated cancer drivers in clinical a) glioblastoma and b) ovarian cancer samples.

Supplementary Table 5

Gene	Number of Cell Lines	Amplification Freq in Ovarian Tumors	Number of shRNAs w/ Reduced Proliferation
**MYC**	2	30.7	4, 4
**KRAS**	2	10.8	4, 3
**BCL2L1**	1	5.1	4
**EVPL**	1	2.5	4
**GRB2**	1	3.5	3
**JUN**	1	2.2	3
MAPK1	1	1.6	3
**MAFG**	2	3.2	2, 2

Table S5. List of 11 cell-line specific cancer drivers (8 unique drivers) predicted by OncoIMPACT that were validated as essential genes for survival and proliferation using shRNA knockdown (see Figure 3b). Bold genes represent cancer drivers that were also predicted by OncoIMPACT in clinical tumour samples.

Supplementary Figure 1

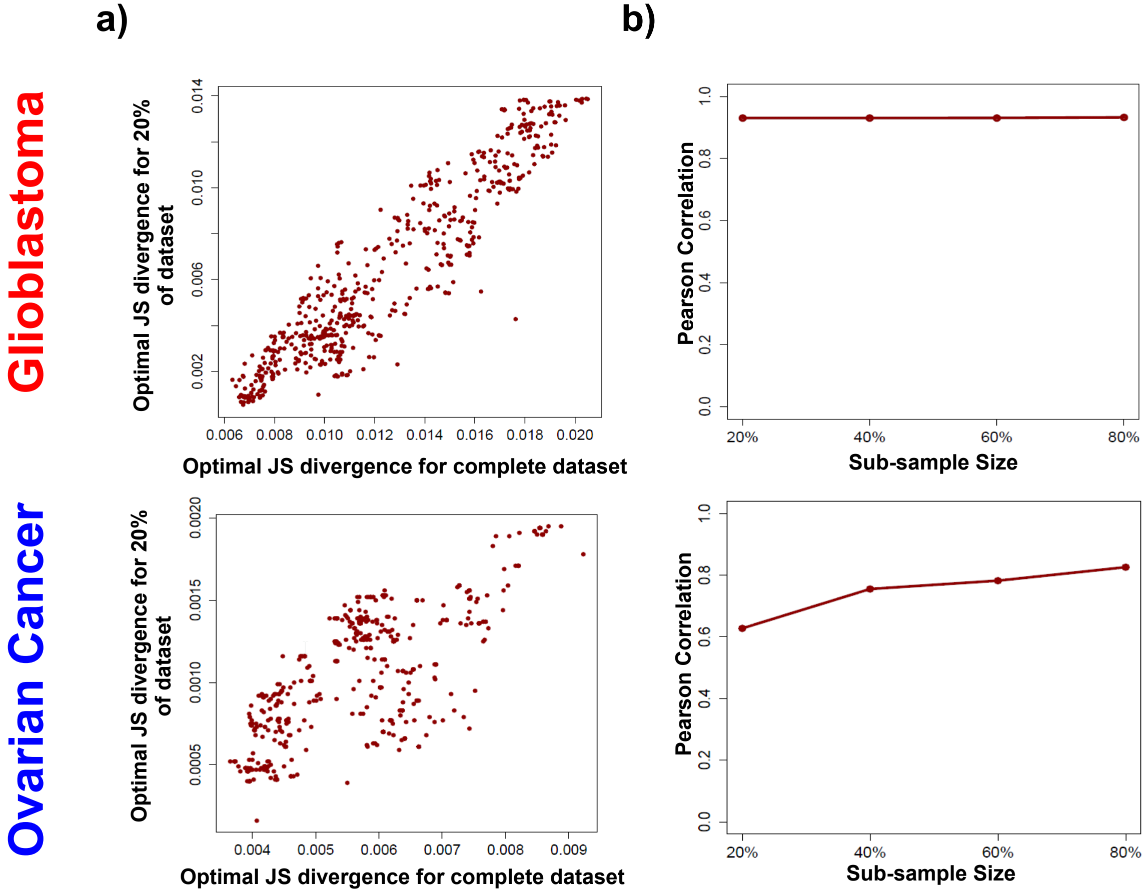

c)

Size	F	L	D
100%	2.5	16	65
80%	2.5	14-20	55
60%	2.5	14-20	55
40%	2.5	14-20	55
20%	2.5	14-20	55

Size	F	L	D
100%	2	4	90
80%	2	4	100
60%	2	4-8	100
40%	2	6-8	100
20%	2	6-8	100

Figure S1. Robustness of the parameter estimation procedure. All results shown are calculated with 100 random sub-samples. (a) Comparison of Optimal Jensen-Shannon (JS) divergence (for different set of parameters) obtained on the complete data set versus using 20% of the data. (b) Pearson correlation of JS divergence values (against those on the full dataset) as a function of the sub-sampling size. (c) Parameter settings (F = Fold change of genes; L = Length of path; D = Degree of nodes) obtained using different sub-sample sizes.

Supplementary Figure 2

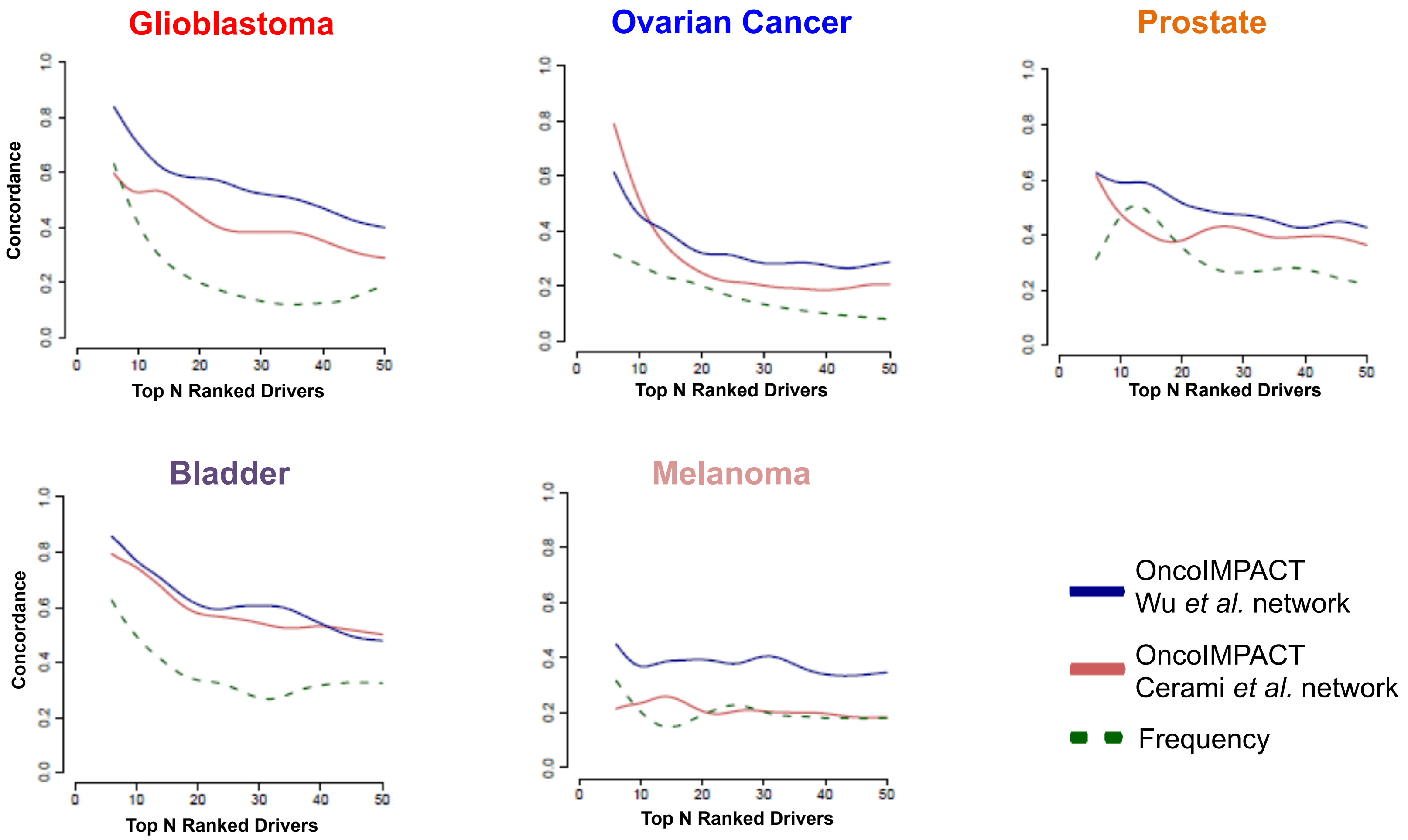

Figure S2. Influence of the interaction network on OncoIMPACT's results. The y-axis shows percentage concordance with cancer gene census and pan-cancer drivers for the top N ranked drivers reported by a method. Note that the network from Cerami et al. is comparable to the one from Wu et al. in terms of the number of genes (9,261 vs 9,452) and overlap with the validation set (78% vs 75%) but has significantly fewer interaction edges (68,102 vs 181,706).

Supplementary Figure 3a

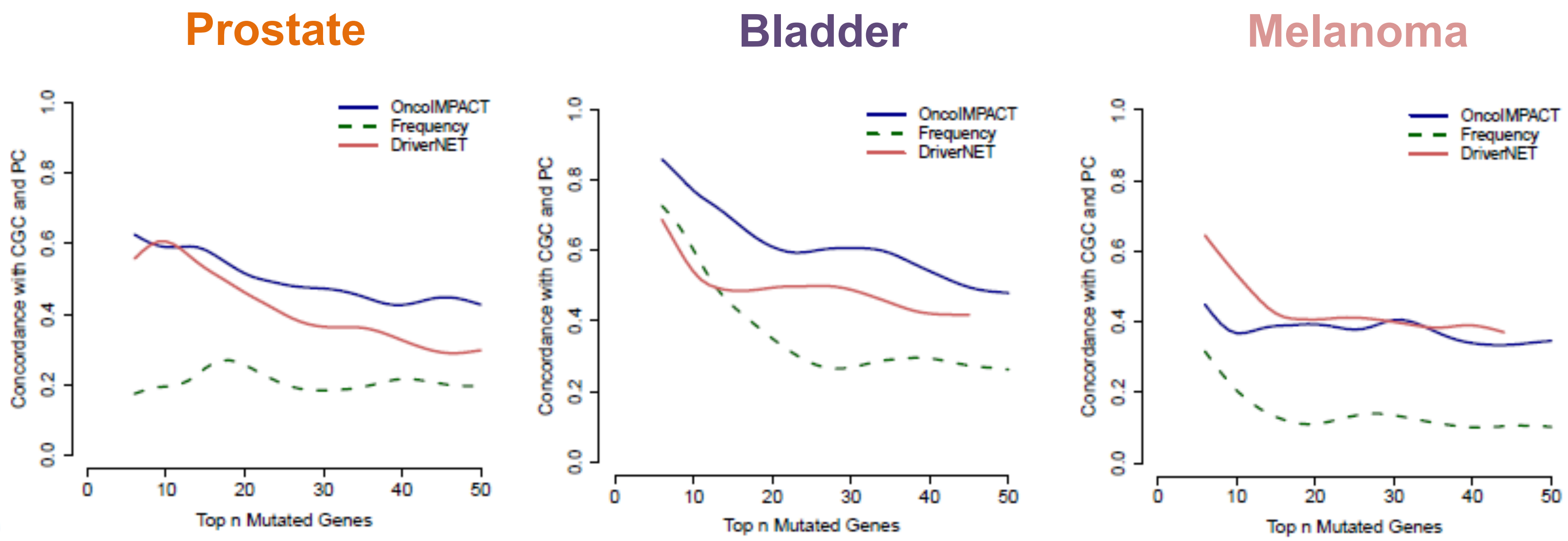

Figure S3a. Percentage concordance with cancer gene census and pan-cancer drivers for the top N ranked drivers from OncoIMPACT, DriverNET and frequency-based predictions on Prostate, Bladder and Melanoma TCGA datasets.

Supplementary Figure 3b

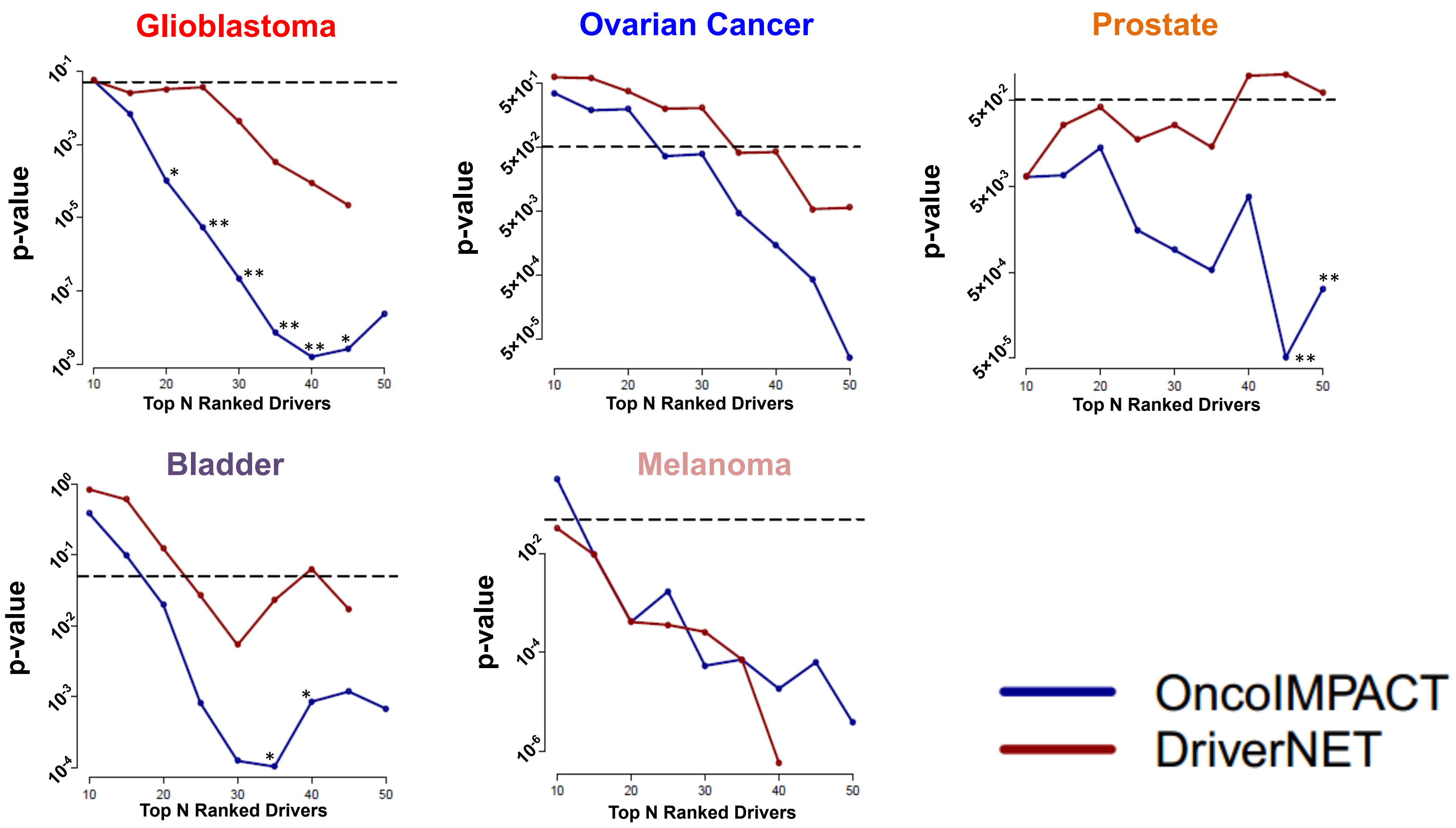

Figure S3b. Enrichment for driver genes (cancer gene census and pan-cancer) in OncoIMPACT and DriverNET over a frequency-based approach. Points where OncoIMPACT's predictions are significantly better than DriverNET are indicated by * (p-value < 0.1) and ** (p-value < 0.05). P-values were computed using a one-sided exact binomial test for an excess of driver genes in the top N predicted drivers for a method when compared to the corresponding frequency for the alternate method.

Supplementary Figure 4

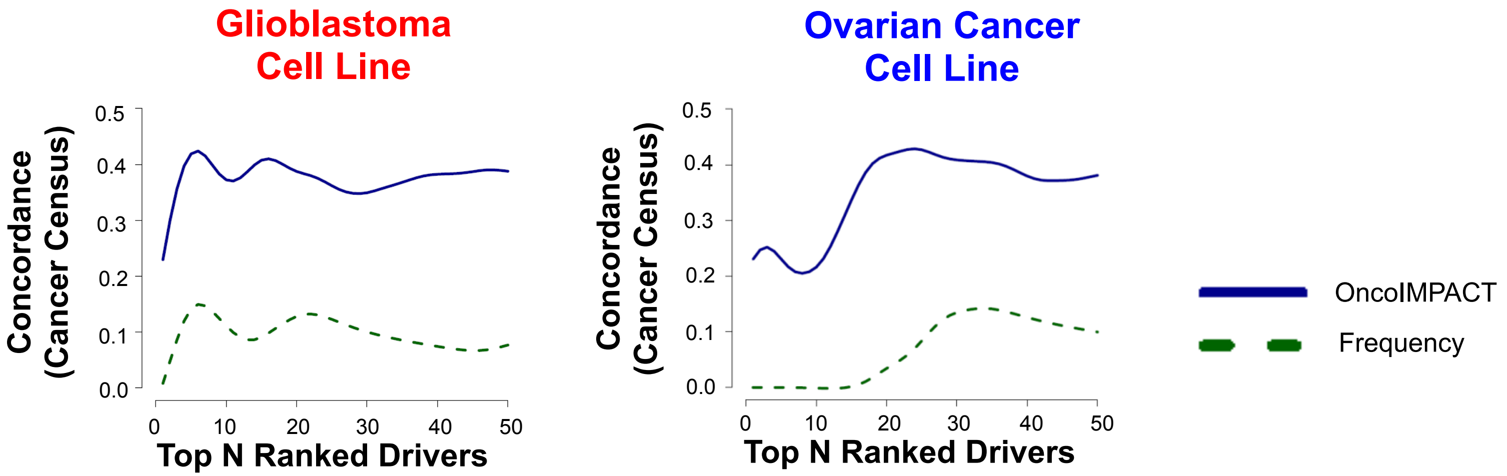

Figure S4. Percentage concordance with cancer census genes for the top N ranked drivers from OncoIMPACT and frequency-based predictions.

Supplementary Figure 5

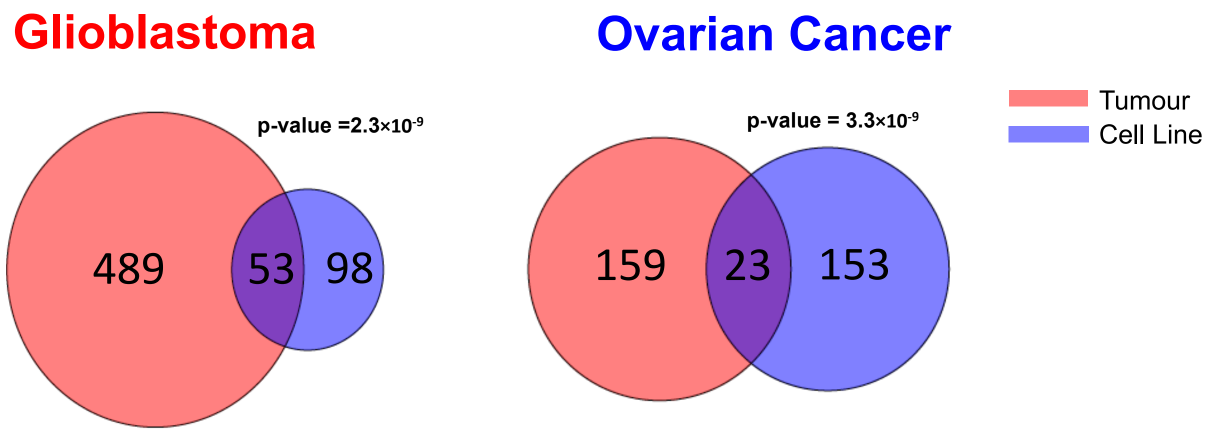

Figure S5. Venn diagrams depicting the overlap between OncoIMPACT-predicted cancer drivers in clinical tumor samples (TCGA) and cancer cell lines (CCLE) for (Left) Glioblastoma and (Right) Ovarian cancer. The *p*-values were computed using the hypergeometric test.

Supplementary Figure 6

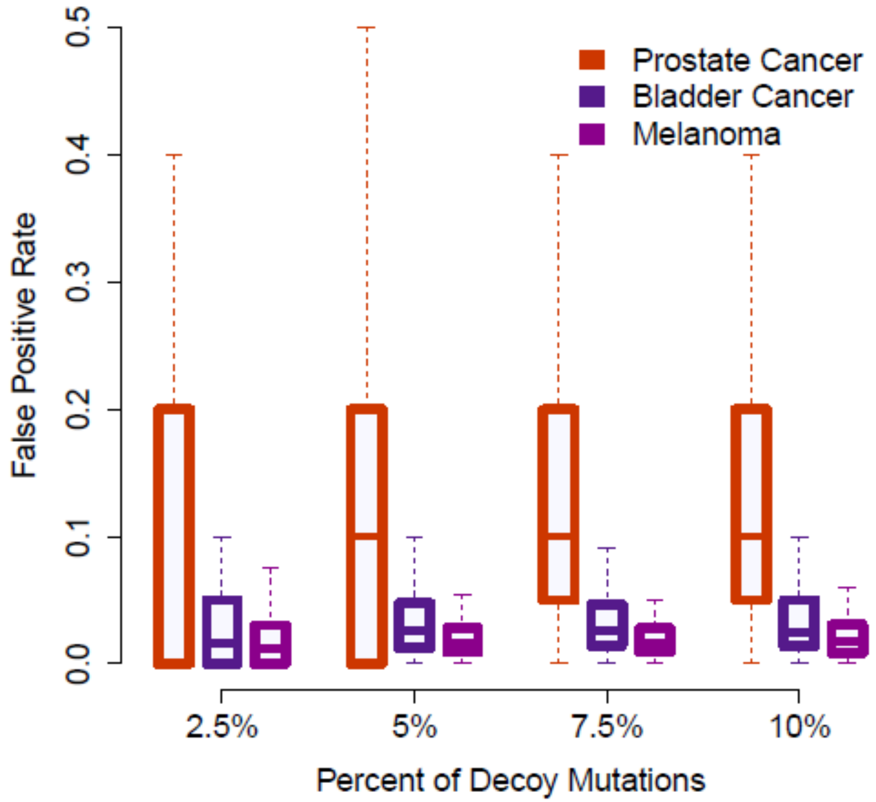

Figure S6. Box plots depicting the distribution across samples of false positive rate for driver gene predictions for Prostate cancer, Bladder cancer and Melanoma in OncoIMPACT. Decoy mutations were introduced in random genes as proxy for non-drivers in this assessment (average of 20 simulations).

Supplementary Figure 7

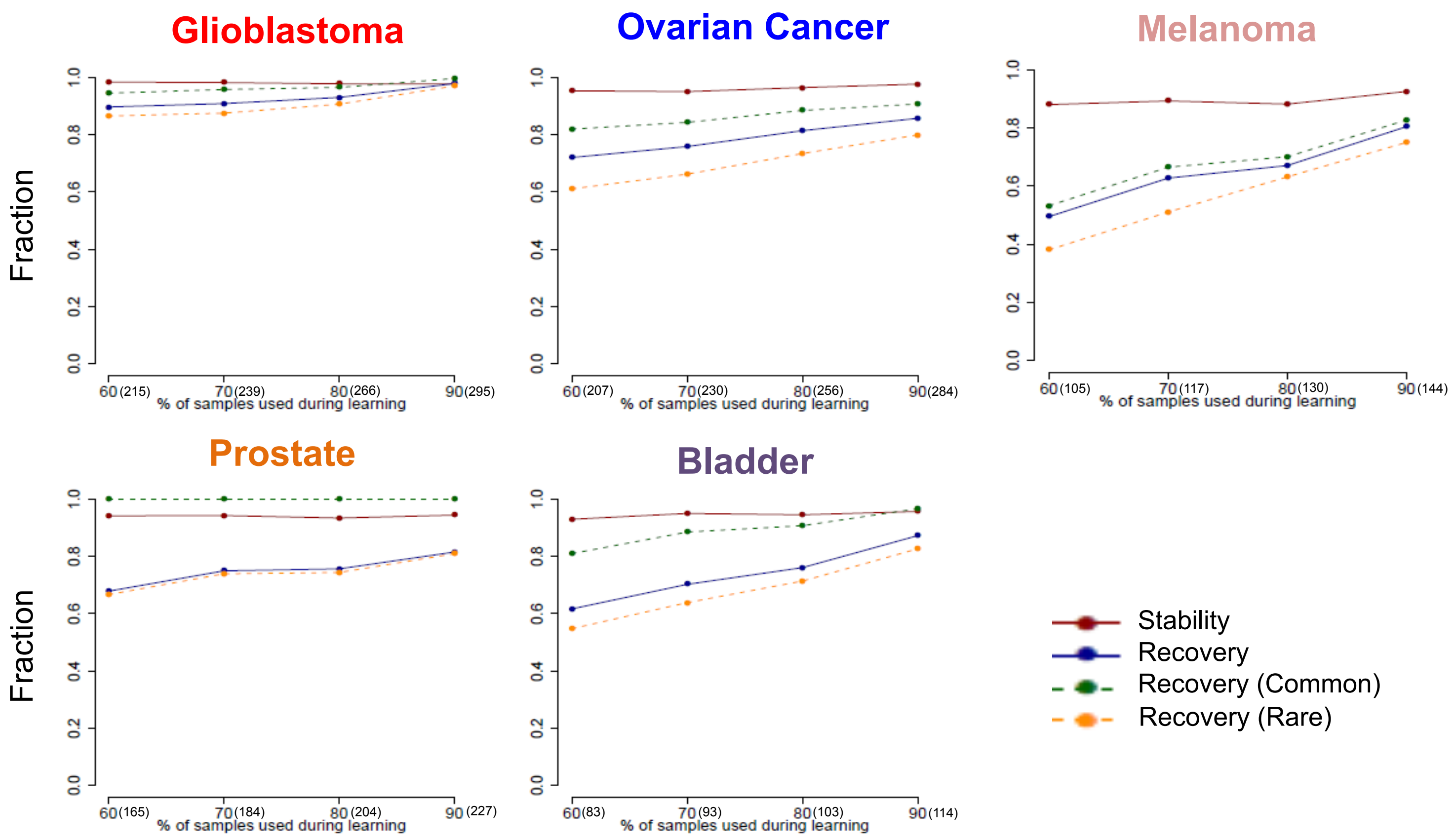

Figure S7. Stability (precision when evaluated on predictions from the full dataset) and recovery (sensitivity when evaluated on the full dataset) characteristics of patient-specific driver gene prediction by OncoIMPACT as a function of the size of the dataset used for learning phenotype genes (average of 5 cross-validation runs). Note that corresponding sample sizes are shown in parenthesis. Prediction was done on the rest of the dataset that was not used for learning phenotype genes.

Supplementary Figure 8

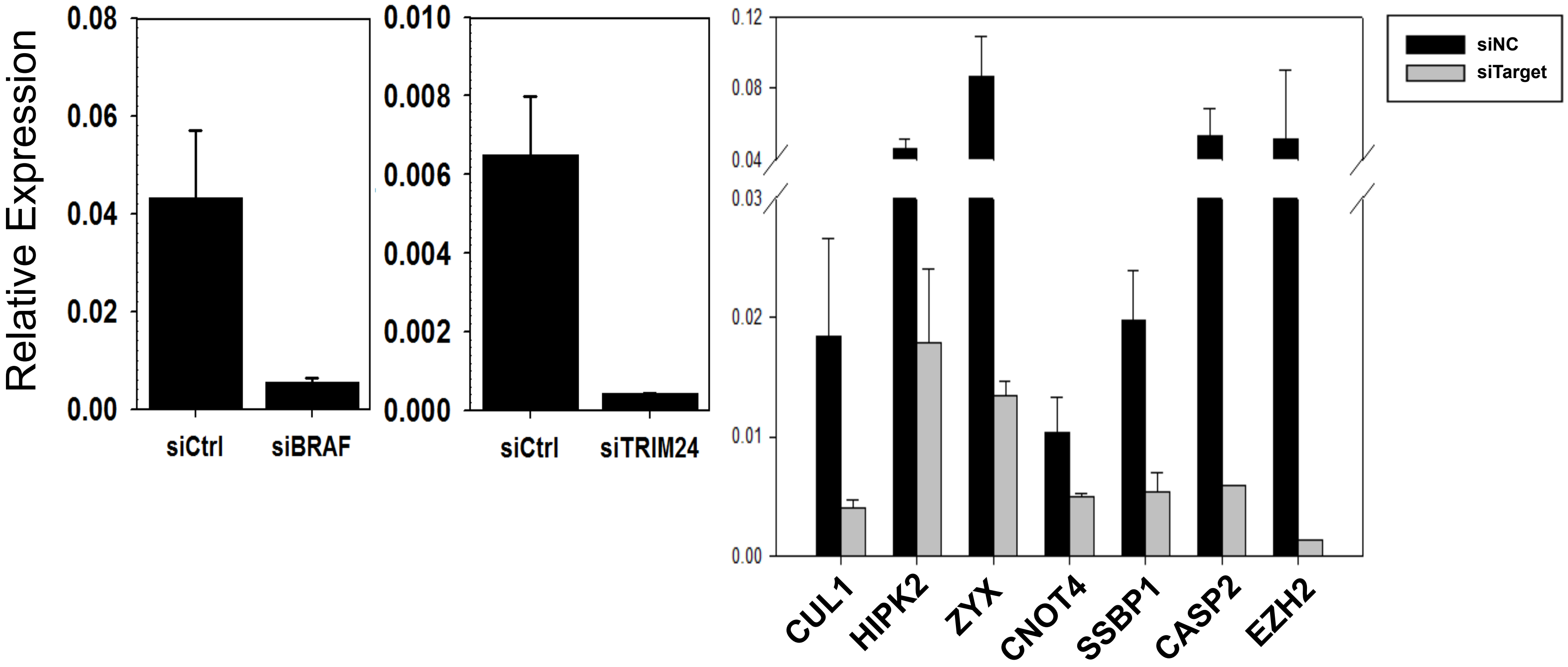

Figure S8. Relative mRNA expression of (Left) BRAF, (Center) TRIM24 and (Right) 7 selected amplified genes (not predicted as drivers by OncoIMPACT) in melanoma cells treated with control siRNA vs siRNA targeting the respective gene. GAPDH serves as an internal normalization control. Error bars represent standard-error of mean (S.E.M.) of at least 2 independent repeats.

Supplementary Figure 9

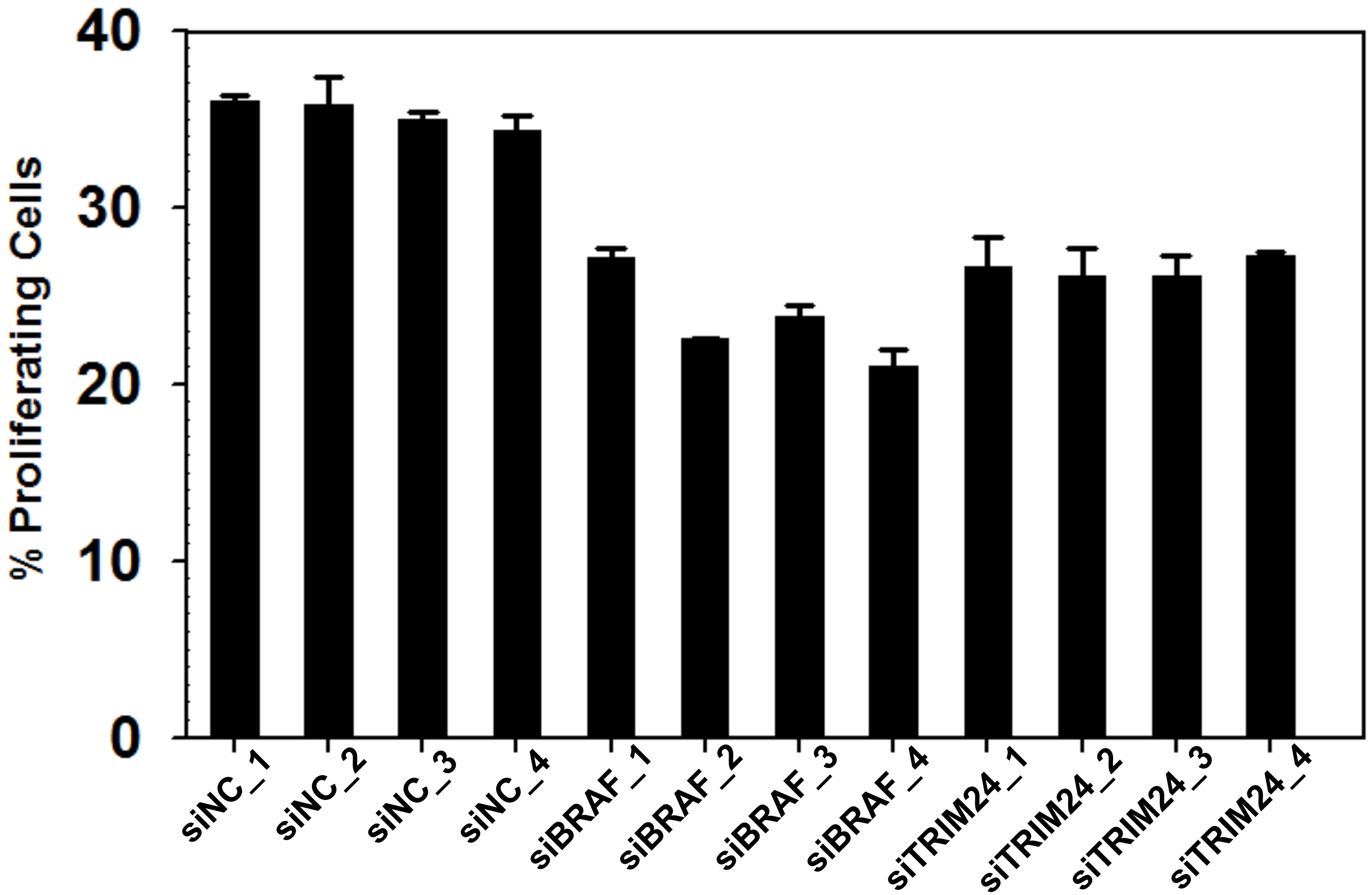

Figure S9. Cell proliferation assay in a patient-derived melanoma cell-line treated with control siRNAs or individual siRNAs targeting BRAF or TRIM24. Error bars represent S.E.M. of 3 independent repeats.

Supplementary Figure 10

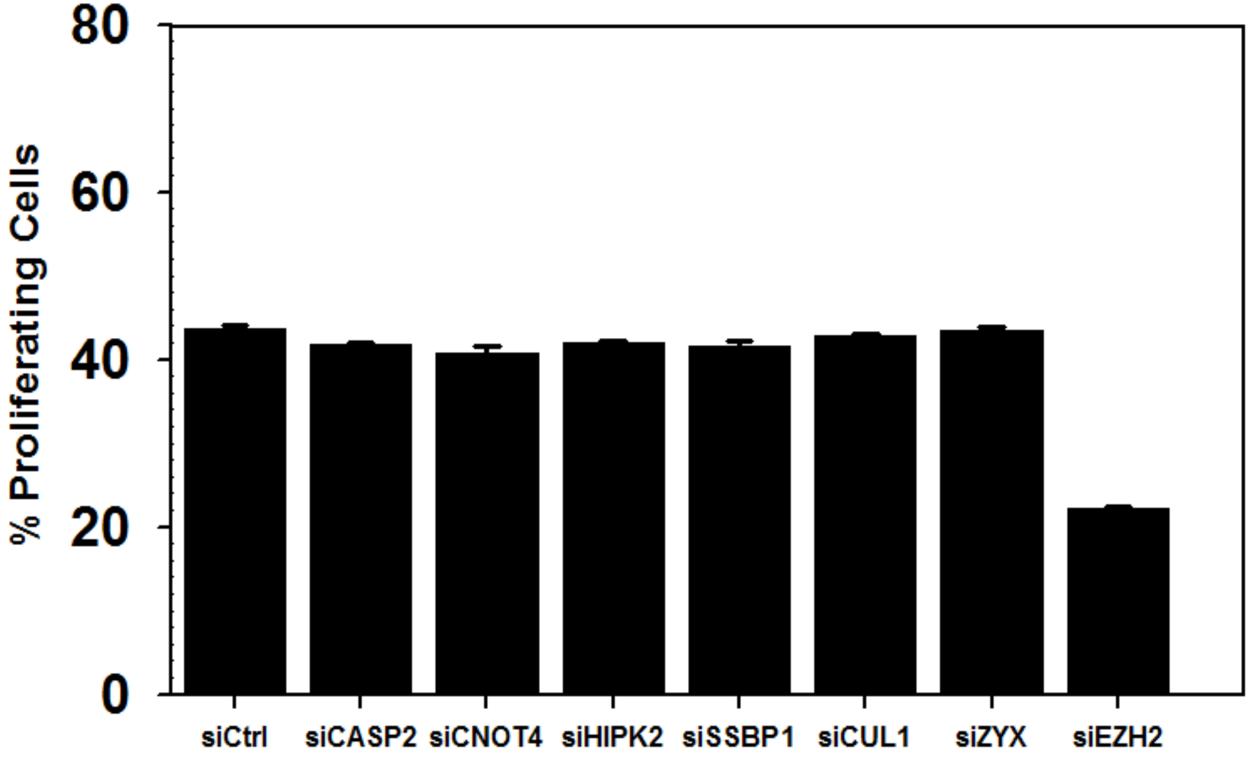

Figure S10. Cell proliferation assay in a patient-derived melanoma cell-line treated with control siRNA or siRNA targeting 7 different selected amplified genes not predicted as drivers by OncoIMPACT but with functional roles in oncogenic processes. Error bars represent S.E.M. of 3 independent repeats.

Supplementary Figure 11

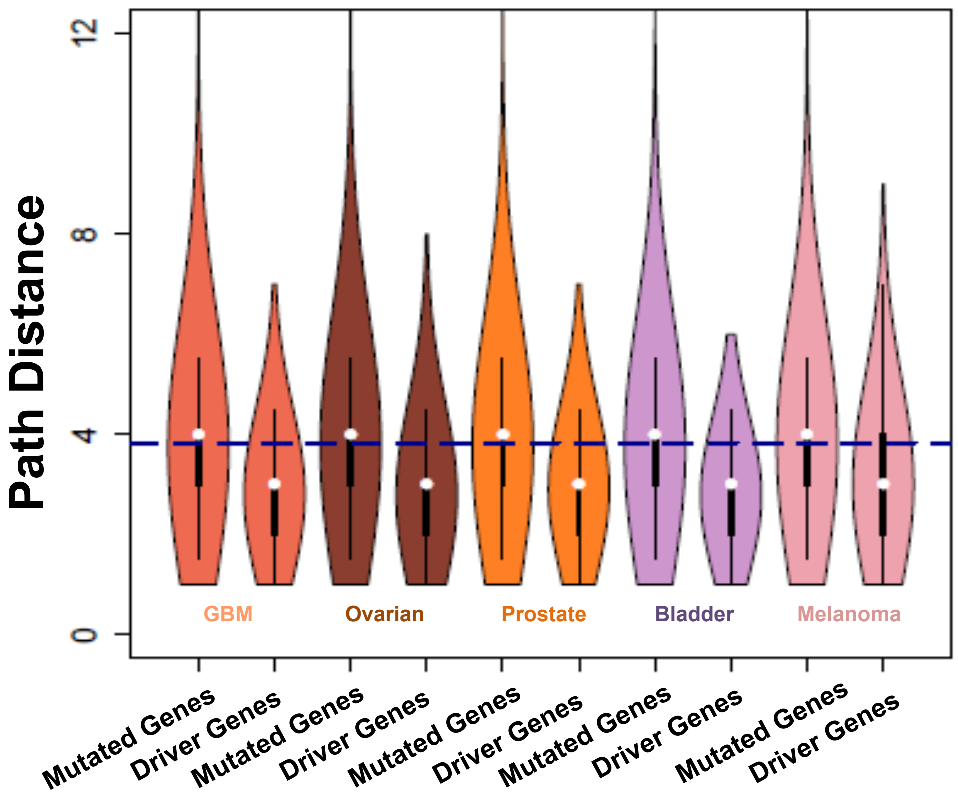

Figure S11. Violin plots showing the distribution of distances in the gene interaction network between all pairs of genes in each class (mutated genes and predicted driver genes at the aggregate level). The blue line represents the average distance between genes on the interaction network. Note that this figure is the analog of **fig. 4a** and **Suppl. fig. 12** without patient-specific analysis.

Supplementary Figure 12

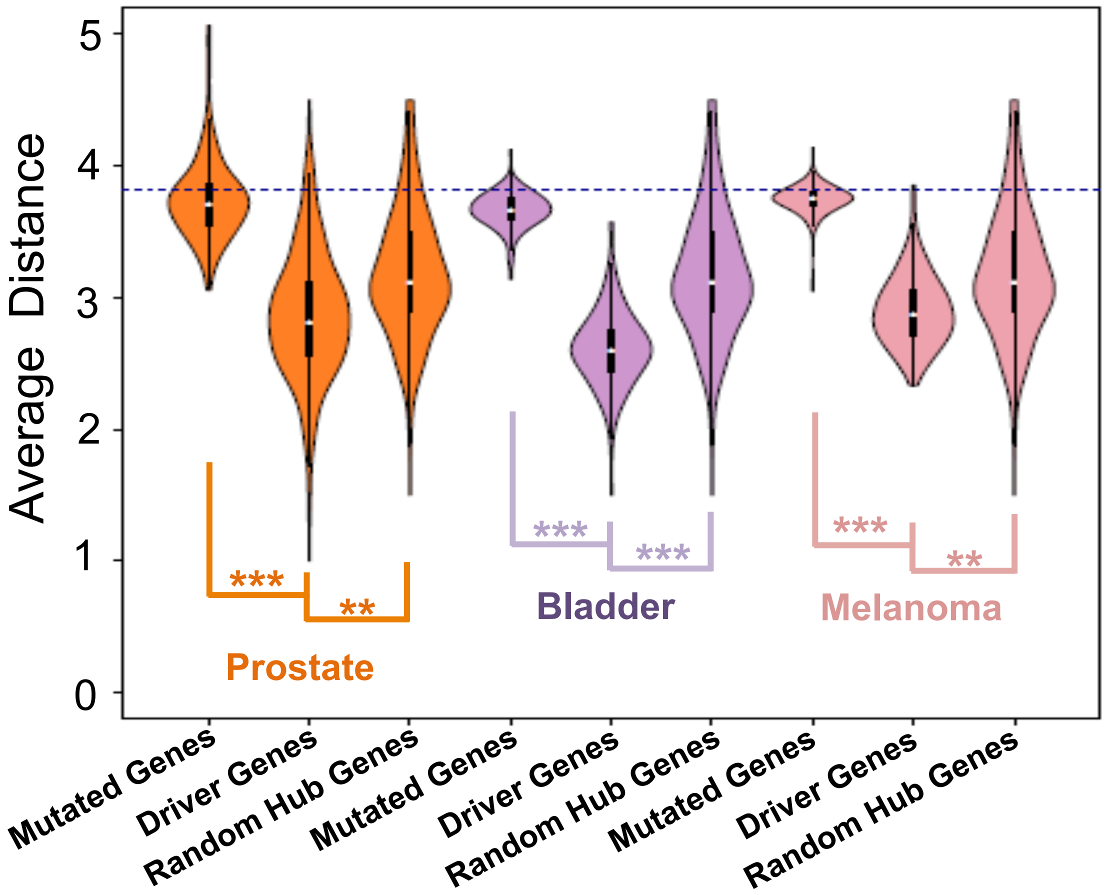

Figure S12. Violin plots showing the distribution of average distance in the gene interaction network (computed at a sample-specific level) between all pairs of genes in each class (mutated genes, predicted driver genes and random hub genes (degree ≥20)). The blue line represents the average distance between genes on the interaction network. The p-values are computed using wilcoxon rank-sum test. ***p-value$<2.2\times10^{-16}$; **p-value$<1.47\times10^{-10}$

Supplementary Figure 13

a)

		GBM	Ovarian	Prostate	Bladder	Melanoma
**No.of Drivers**	**OncoIMPACT**	547	182	691	520	517
	**Frequency (>=5%)**	96	1082	14	547	2844
**No.of Co-drivers**	**OncoIMPACT**	1033 (8.3%)	200 (3.5%)	257 (7%)	8 (0.01%)	8 (0.05%)
	**Frequency (=>5%)**	521 (12%)	48398 (9.1%)	3 (4%)	99 (2%)	125340 (3.4%)

b)

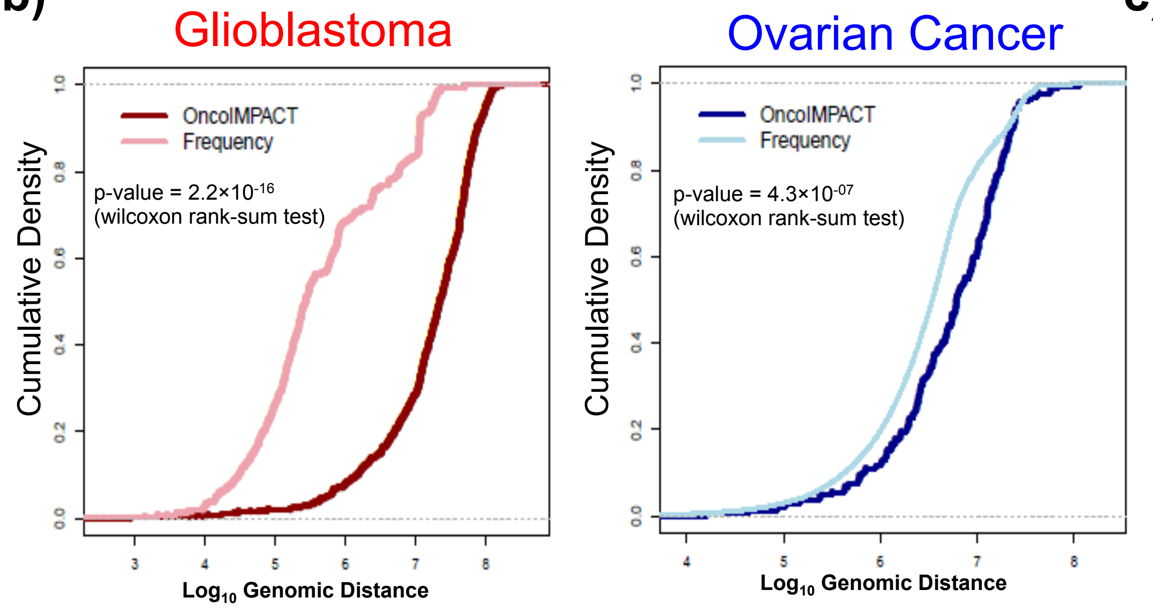

c)

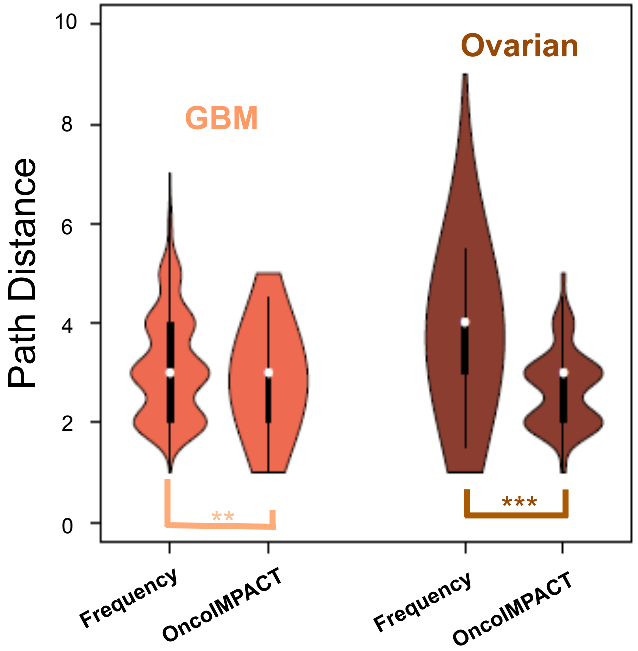

Figure S13. a) Table showing number of significant (hypergeometric test) co-driver pairs. The frequency based method considers all mutations with a frequency higher than 5% to be drivers. Numbers in parenthesis represent the percentage of tested pairs that are significant. b) Distribution of genomic distance of co-driver pairs. c) Violin plots showing the distribution of distances on the gene interaction network (proxy for functional similarity) between co-driver pairs. The p-values are computed using wilcoxon rank-sum test. ***p-value < 2.2×10^{-16}; **p-value = 3.2×10^{-11}

Supplementary Figure 14

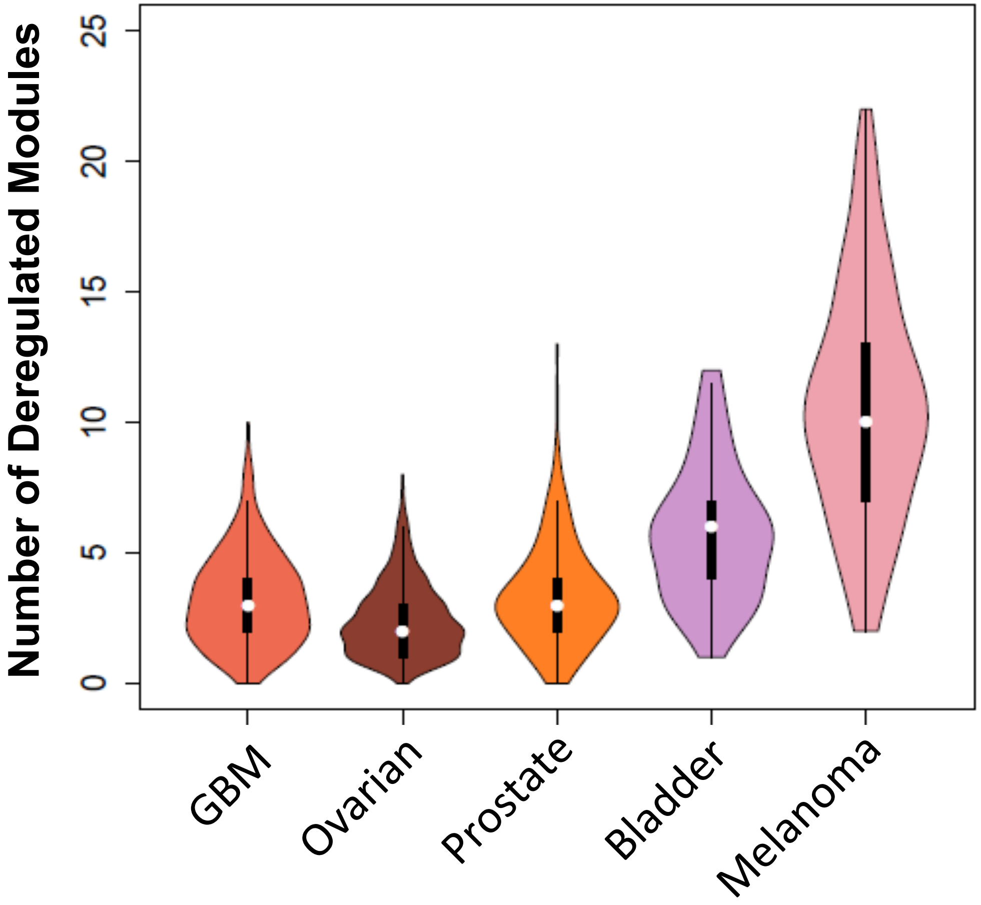

Figure S14. Violin plots showing the distribution of the number of deregulated modules per patient for a variety of cancers.

Supplementary Figure 15

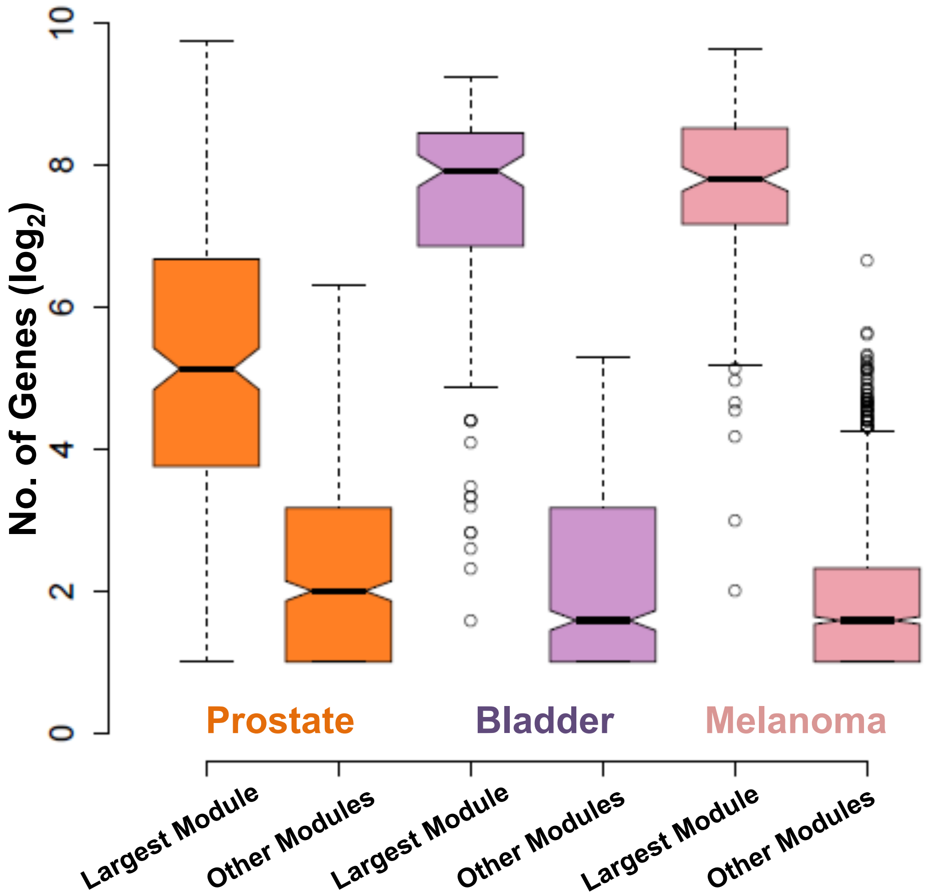

Figure S15. Box plots depicting the distribution of the number of genes in the largest module and all other modules.

Supplementary Figure 16

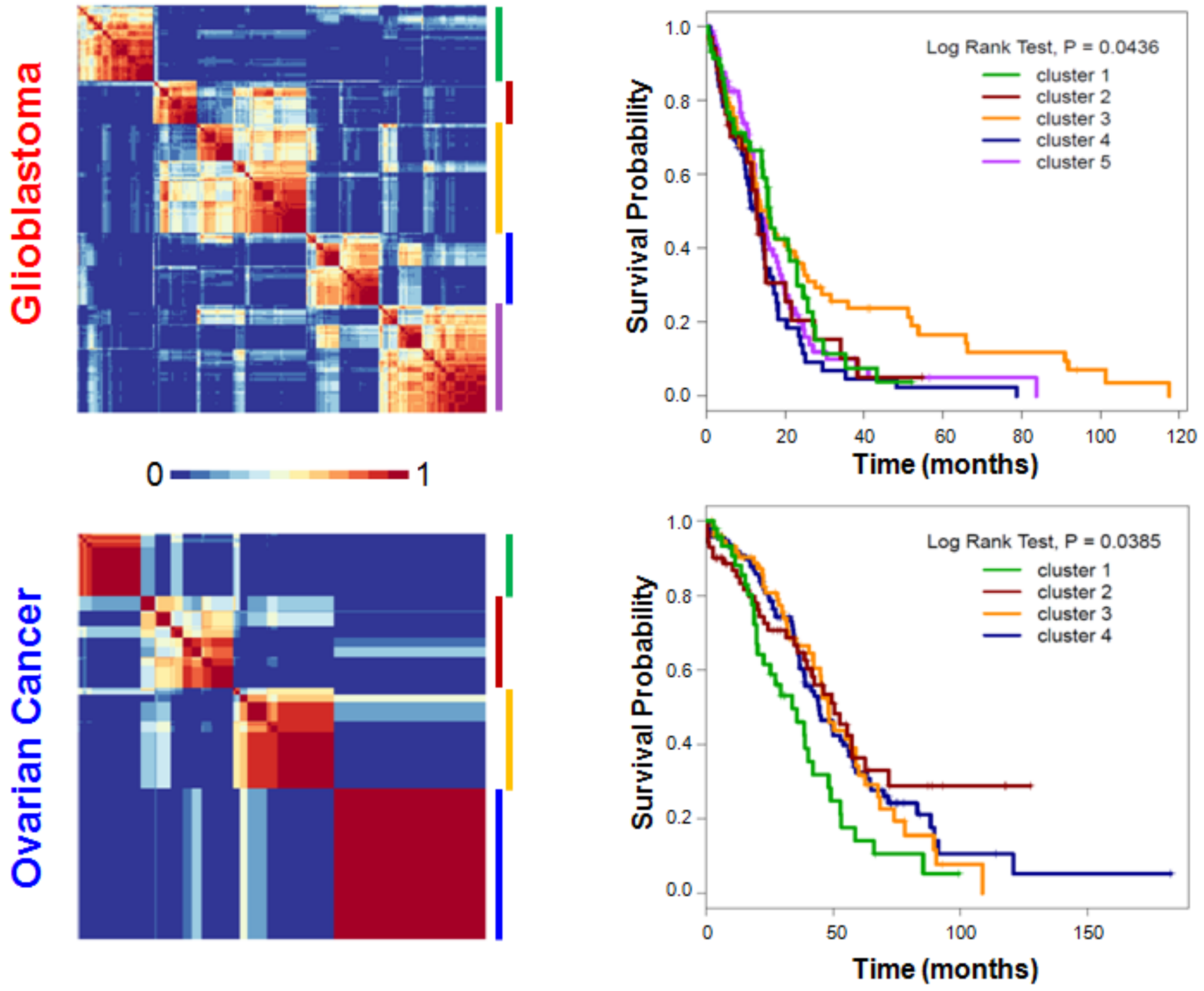

Figure S16. (Right Panel) NMF consensus clustering of glioblastoma and ovarian cancer patients using the mutational profiles of selected top OncoIMPACT nominated cancer drivers (47 drivers for gliobastoma and 6 drivers for ovarian cancer). **(Left Panel)** Survival profiles of glioblastoma and ovarian cancer patients stratified by the accompanying consensus clustering.

Supplementary Figure 17

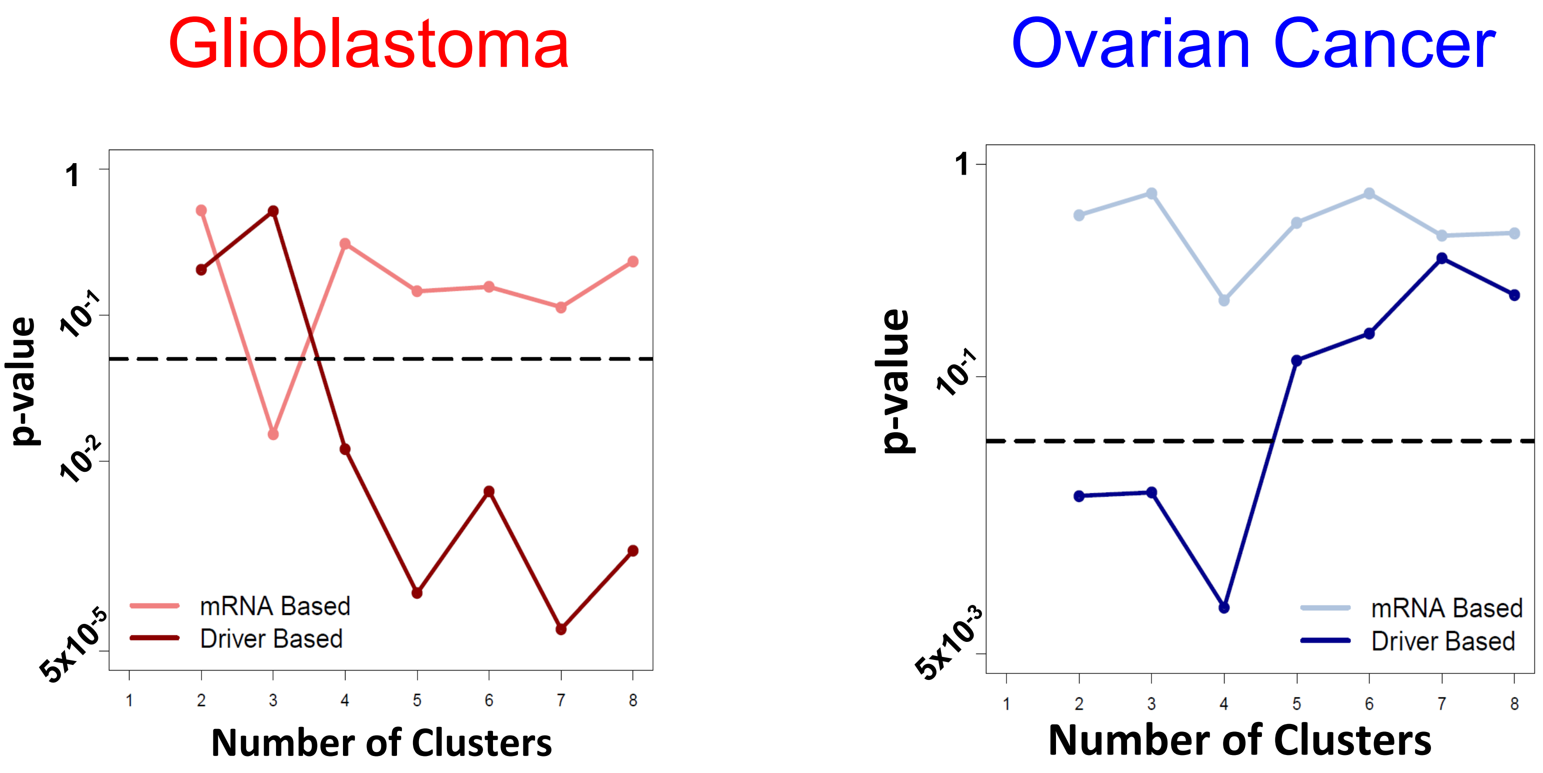

Figure S17. Comparison of mRNA- and driver- based stratification of patients (measured by Log Rank Test *p*-value for patient survival profiles across clusters) as a function of the number of clusters.

Supplementary Figure 18

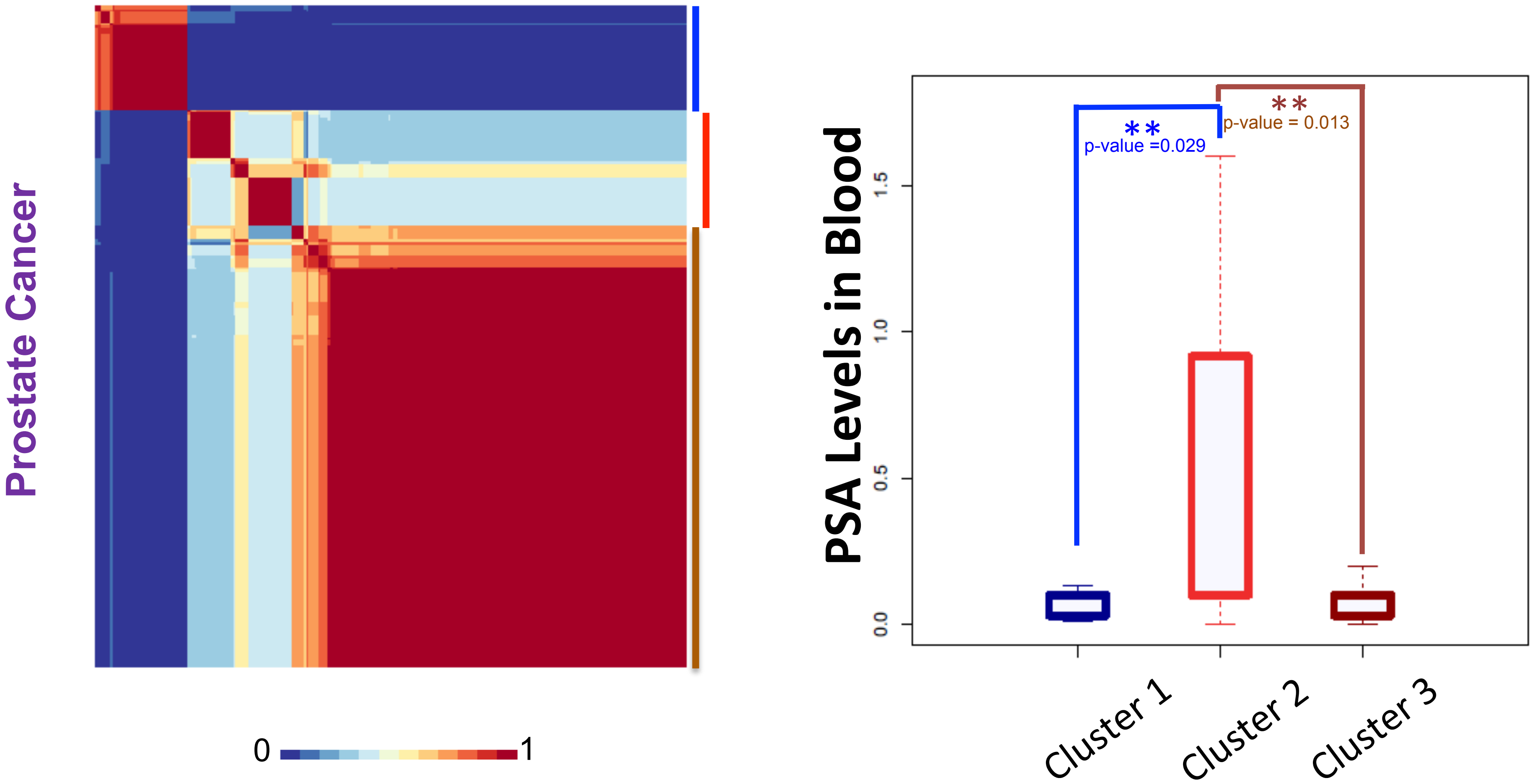

Figure S18. (Left Panel) NMF consensus clustering of prostate cancer patients using the mutational profiles of selected top OncoIMPACT-nominated cancer drivers (31 drivers). **(Right Panel)** PSA blood levels of prostate cancer patients stratified by the accompanying consensus clustering. The *p*-values were computed using the wilcoxon rank-sum test.
