# Supplementary material for: Patient-specific driver gene prediction and risk assessment through integrated network analysis of cancer omics profiles
Source: Nucleic Acids Res. 2015 Jan 8;43(7):e44. doi: 10.1093/nar/gku1393 (PMC4402507; doi:10.1093/nar/gku1393)
Supplement: SUPPLEMENTARY DATA [file supp_gku1393_nar-02721-met-f-2014-File008.pdf]

Supplementary Text

TCGA data analysis

SNV analysis

SNV data (level 2) was obtained from the TCGA website (https://tcga-data.nci.nih.gov). For our analysis, we only considered indel, missense and nonsense mutations (i.e. all non-synonymous mutations).

CNV analysis

The CNV data for Ovarian Cancer and Melanoma were obtained from the CBIO portal (http://www.cbioportal.org/public-portal/). Genes with a GISTIC prediction of 2 or -2 were considered as amplified or deleted, respectively. For other cancer types, we downloaded segment copy number values (level 3) from the TCGA website (https://tcga-data.nci.nih.gov). Segments with a mean copy number value greater than 1 or less than -1 were considered amplified or deleted and all genes within a segment were considered mutated. For sex chromosomes we applied the following rules: CNV events on chromosome Y of female samples were discarded; for male samples, copy number change values were adjusted (by adding 0.5 to the values) to account for a single copy of chromosomes X and Y.

Expression Array analysis (Glioblastoma and Ovarian Cancer)

Expression values were downloaded from the TCGA website (level 3; https://tcga-data.nci.nih.gov) for the normal and the tumor samples. All normal samples provided in the TCGA web site were used as controls (10 for Glioblastoma and 7 for Ovarian Cancer). For each tumor sample, log2 fold change for each gene was computed by comparing to the median log2 expression value for the gene in normal samples. Fold-change significance was assessed using a t-test. Genes with a FDR corrected p-value below 0.05 were reported as deregulated for analysis with OncoIMPACT (sensitive mode).

RNA-seq analysis (Melanoma, Prostate and Bladder Cancer)

Raw read count values for cancer and normal samples were downloaded from the TCGA website (level 3; https://tcga-data.nci.nih.gov). Due to the presence of a large number of normal controls for some cancer types, we selected 7 normal samples for Prostate Cancer (TCGA-G9-6333-11A-01R-1965-07; TCGA-HC-7211-11A-01R-2118-07;TCGA-HC-7737-11A-02R-2118-07; TCGA-HC-7738-11A-01R-2118-07; TCGA-HC-7740-11A-01R-2118-07; TCGA-HC-7745-11A-01R-2118-07; TCGA-HC-7747-11A-01R-2118-07; TCGA-HC-8258-11A-01R-2263-07) and 7 for Bladder Cancer (TCGA-GC-A3WC-11A-11R-A22U-07; TCGA-CU-A0YR-11A-13R-A10U-07; TCGA-BT-A20N-11A-11R-A14Y-07; TCGA-BT-A20R-11A-11R-A16R-07; TCGA-CU-A0YN-11A-11R-A10U-07; TCGA-BL-A13J-11A-13R-A10U-07; TCGA-BT-A20Q-11A-11R-A14Y-07) to serve as controls for the differential expression analysis. We use DESeq2 (1) to perform the analysis. We normalized the read count of the whole data set (controls and cancers samples were pooled together). For the Prostate and Bladder Cancer datasets, we computed differential gene expression between each cancer sample and the set of normal controls using their respective normalized read counts using DESeq2. All genes with a FDR value below 0.05 and average read count > 200 (to avoid noise from lowly expressed genes) were reported as deregulated (no fold-change cutoff) for OncoIMPACT analysis (stringent mode). For Melanoma, as only one normal control sample was available, we log transformed the data (using “rlogtransfom” in DESeq2) to compute fold-changes and genes with fold-change > 1 and average read count > 200 were selected as deregulated.

DriverNET analysis

Gene expression values (array datasets) and normalized reads counts (RNA-seq datasets) were downloaded from the TCGA website (level 3; https://tcga-data.nci.nih.gov).

Introduction of decoy mutations

For each sample, decoy mutations were added on random genes that belong to the interaction network and that are not mutated for that particular sample. The number of decoy mutations was computed for each sample s using the following formula: round($r \times m_s$), where r represents the decoy mutation rate and m_s the number of mutations in sample s.

CCLE data analysis

SNV analysis

We downloaded the SNV list with no common SNPs and no neutral variants from the CCLE website (http://www.broadinstitute.org/ccle/home). Every point mutation/indel with a frequency in the CCLE data set greater than 4.5% (highest frequency observed for a point mutation in the TCGA ovarian cancer dataset i.e. for TP53:7517845) was filtered out as a potential germline mutation.

CNV calls

CNV calls were collected using the same filters as were used for the TCGA datasets.

Expression array differential expression analysis

We used normal samples from TCGA to perform differential expression analysis. Specifically, we downloaded CEL files from TCGA (level 1; https://tcga-data.nci.nih.gov) and CCLE web sites (http://www.broadinstitute.org/ccle/home) and probe expression values were computed using the R library "affy" and quantile normalized using the R library "preprocessCore". Differential expression analysis was performed at the probe level using the same steps as described in a previous section. Genes with significant probe fold changes in opposite directions were discarded. Fold changes of the probe with the smallest p-value were reported for a gene.

Achilles data analysis

OncoIMPACT analysis

We used the Ovarian TCGA dataset to determine parameters and phenotypes using OncoIMPACT's database mode. The 47 ovarian cancer cell-lines were then analyzed using OncoIMPACT's discovery mode.

shRNA analysis

We downloaded shRNA level log fold-change data from the Achilles website (http://www.broadinstitute.org/achilles). Specific shRNAs with log fold-change scores below -1 were considered as significantly reducing cell proliferation.

Genomic analysis of melanoma sample

Exome Sequencing

3ug of genomic DNA was sheared. With ampure beads, the sheared DNA was purified and sub-150bp fragments were removed. The size and quality of the sheared DNA was assessed with agilent DNA 1000 assay. End-repair was done using NEBNext End Repair kit. After dA-tailing and paired-end adapter ligation, amplification was carried out. Roche NimbleGen protocol was used for hybridization of the amplified sample library and the exome library. The thermocycler's heated lid should be turned on and set to maintain 57°C (+10°C above the hybridization temperature). Next, washing and recovery of the captured DNA from the hybridization of the amplified sample library and exome library was done using Streptavidin Dynabeads. Then, the captured DNA, bound to the Streptavidin Dynabeads, was amplified using LM-PCR. To minimize PCR bias, 2 reactions per sample were performed and subsequently combined. The amplified captured DNA was cleaned up using Qiagen QIAquick PCR Purification Kit. The amplified captured DNA met the required criteria: the A260/A280 ratio measured by NanoDrop. In short, COT DNA and PE-HE1 and PE-HE2 Oligos (PE =

Paired End; HE = Hybridization Enhancing) were added to the amplified sample library and then the component was dried in a DNA vacuum concentrator on high heat (60°C). After adding the Hybridization Cocktail (2X Hybridization Buffer + Hybridization Component A), the amplified sample library/COT DNA/PE-HE Oligos/Hybridization Cocktail was transferred to the 4.5 µl aliquot of Exome Library and incubated in a thermocycler at 47°C for 64 - 72 hours. Spectrophotometer was 1.7 - 2.0; the LM-PCR yield was more than 1.0 µg; and the average fragment length was 150 - 400 bp. The negative control did not show significant amplification, indicative of no contamination. The amplified captured DNA was sent for sequencing after ensuring that the enrichment measured by qPCR was successful: for this assay two internal control loci were tested; these loci were included as capture targets in SeqCap EZ Exome Library. Relative quantification comparing the control targets in amplified sample library (pre-hybridization) and the corresponding amplified captured DNA (post-hybridization) was done using raw Cp values (obtained in qPCR assay) to check the fold enrichment. All samples with successful enrichment (fold enrichment>50) generated higher Cp value for pre-hybridization compared to post-hybridization. Moreover, the melting curve (dissociation) analysis verified that there was not any nonspecific contribution to the Cp values for any samples.

Paired-end reads were generated using an Illumina sequencer and mapped to the reference human genome (UCSC hg19) using ELAND (v1.8.2, default parameters) (Illumina Inc.). Reads that failed the quality control filter were removed from further analysis. The mapping was further refined by applying the realignment module of GATK (v1.0.5974, default parameters) (2). Finally base quality values were re-calibrated using GATK (default parameters). SNVs were called for each sample (tumor and normal) separately using SAMtools (v0.1.17, SNP-quality threshold = 20, Consensus-quality threshold = 30) (3). Variant calls specific to the tumor for which the genotype in the normal pair was equal to the reference genome were selected as somatic variants.

Illumina Paired-end RNA-seq

Prior to library construction, the integrity of the RNA sample was checked using Agilent RNA Pico 6000 and samples with RNA Integrity Number (RIN) of value ≥ 8.0 were used for library construction. Using Illumina TruSeq™ RNA Sample Preparation Kit, cDNA libraries were prepared in accordance with manufacturer's protocol and sent for 2×50 paired-end Illumina sequencing.

Paired-end Illumina reads was mapped to the reference human genome (UCSC hg19) using Tophat (v1.4.1, butterfly search, 2 transcription mismatches allowed) (4). We compare the gene expression of the tumor with a set of 3 normal melanocyte samples using the Cuffdiff software (v2.0.1, default parameters, gene annotation from RefSeq) (4). Genes with a log2 fold change higher than 1 and a corrected p-value < 0.1 were considered deregulated.

DNA-PET: library construction and quality assessment

2x50bp mate-pair libraries were sequenced on the SOLiD™ platform. This mate-paired library originates from the two ends of the same genomic DNA fragment. To obtain 10kb fragments, the HydroShear DNA Shearing Device (setting: large shearing assembly at speed code=10) was used to shear the genomic DNA (starting DNA amount is at least 30ug). Then, LMP CAP linkers (ABI SOLiD oligos kit) were ligated to both ends of the end-repaired sheared DNA. To excise the gel with 10kb DNA fragment size and fragment *selection* at 1kb *intervals*, gel electrophoresis was performed using 0.5% agarose gel at 80V for 20min, followed by 20V for 22-24 hours at room temperature. DNA extraction from the 10kb excised fragments was performed using QIAEXII Gel Extraction Kit. To choose an appropriate insert DNA with correct size, high purity and enough concentration for circularization, Agilent DNA 12000 assay was performed on size-selected DNA fragments (7 to 11kb DNA). Minimum amount of 500ng DNA (insert DNA) was used for circularization with internal adaptors. The LMP CAP Adaptor did not have the 5′ phosphate in one of its oligonucleotides, therefore the resulting DNA circle had one nick in each strand. Nick translation of circularized DNA was done using *E. coli* DNA Polymerase I, which pushed the nick into the genomic DNA region in 5′ to 3′ direction. T7 exonuclease and S1 nuclease digestion cut the DNA at the position opposite to the nick and released the DNA paired-end tag (PETs) constructs (50bp tags). End-repaired DNA was bound to Streptavidin beads. Then SOLiD Adaptors P1 and P2 (SOLiD Oligos kit)

were ligated to the ends of the mate-paired library for subsequent amplification by PCR. Large scale PCR was performed with adjusted number of PCR cycles according to the intensity of the DNA libraries during quality check PCR trial. DNA Libraries were purified and run on 6% TBE PAGE gel with 25 bp DNA Ladder (Invitrogen) at 200V for 35 minutes. After staining with SYBR Green for 15 min, gel was viewed on Dark Reader to excise the 250-350bp DNA libraries. The gel-purified library was quantified by Agilent DNA 1000 assay to access the purity, size and concentration of the final library. The final suitable libraries were processed using the SOLiD sequencing platform and 50 bp paired-end-tags were generated. Up to 200x genome-wide physical (fragment) coverage was provided for each library sequenced by this method.

Identification of copy number variations

Raw data processing and identification of copy number variations (CNVs) was performed as described previously (5). Briefly, sequenced paired-reads were mapped back to the reference genome hg19, library span range (library insert size) was determined and duplicate PETs were removed. Paired-reads were classified into either concordant PETs (cPETs) or discordant PETs (dPETs). cPETs were the pair end tags which mapped to the reference sequence as expected (both ends map to the same chromosome, and the same strand in the 5'->3' orientation, and within expected distance) representing the consistency between the cancer genome and the reference genome. cPETs were used to detect copy number variations. To define copy number variations (CNVs) the density of cPETs along the entire genome was computed. Genes with a CNV value higher than 1.5 or below 0.7 were considered as amplified or deleted.

OncoIMPACT analysis

To compare each cancer sample to the unique normal control provided by the TCGA website, we log transformed the data (using "rlogtransfom" in DESeq2) to compute fold-changes, and genes with fold-change > 1 and log2 average expression > 7 (to avoid noise from lowly expressed genes) were selected as deregulated. We then used OncoIMPACT's database mode to determine parameters and phenotype genes on the Melanoma TCGA dataset. The in-house melanoma sample was then analyzed using OncoIMPACT's discovery mode.

Distributional properties of driver genes and associated deregulated modules

Gene distance computation

We computed the shortest distance between each pair of genes in the network. Pairs that were unconnected were not taken into account for this analysis.

Generation of sample specific random hub genes

For each sample we simulated a random gene set composed of genes with a degree higher than 20, with size equal to the number of drivers predicted by OncoIMPACT for that sample.

Module annotation

Module annotation was performed using the DAVID perl API (http://david.abcc.ncifcrf.gov) using the category "GOTERM_BP_FAT". Terms with a q-value less than 0.05 were considered as significant.

Co-driver analysis

For each pair of driver genes we assessed the significance of their co-occurrence using the hypergeometric test. Pairs with a FDR below 0.05 were considered as significant. We tested if co-driver gene pairs where also co-mutated using the hypergeometric test and a *p*-value cutoff of 0.05.

References

1. Love, I.M., Huber, W. and Anders, S. (2014) Moderated estimation of fold change and dispersion for RNA-Seq data with DESeq2. *biorxiv*.
2. McKenna, A., Hanna, M., Banks, E., Sivachenko, A., Cibulskis, K., Kernytsky, A., Garimella, K., Altshuler, D., Gabriel, S., Daly, M. *et al.* (2010) The Genome Analysis Toolkit: a MapReduce framework for analyzing next-generation DNA sequencing data. *Genome Res*, **20**, 1297-1303.
3. Li, H., Handsaker, B., Wysoker, A., Fennell, T., Ruan, J., Homer, N., Marth, G., Abecasis, G. and Durbin, R. (2009) The Sequence Alignment/Map format and SAMtools. *Bioinformatics*, **25**, 2078-2079.
4. Trapnell, C., Roberts, A., Goff, L., Pertea, G., Kim, D., Kelley, D.R., Pimentel, H., Salzberg, S.L., Rinn, J.L. and Pachter, L. (2012) Differential gene and transcript expression analysis of RNA-seq experiments with TopHat and Cufflinks. *Nat Protoc*, **7**, 562-578.
5. Yao, F., Ariyaratne, P.N., Hillmer, A.M., Lee, W.H., Li, G., Teo, A.S., Woo, X.Y., Zhang, Z., Chen, J.P., Poh, W.T. *et al.* (2012) Long span DNA paired-end-tag (DNA-PET) sequencing strategy for the interrogation of genomic structural mutations and fusion-point-guided reconstruction of amplicons. *PLoS One*, **7**, e46152.
